# Association among starch storage, metabolism, related genes and growth of Moso bamboo (*Phyllostachys heterocycla)* shoots

**DOI:** 10.1186/s12870-021-03257-2

**Published:** 2021-10-20

**Authors:** Jiajia Zhang, Ruixiang Ma, Xingcui Ding, Manchang Huang, Kai Shen, Siqi Zhao, Zizhang Xiao, Chengming Xiu

**Affiliations:** 1 China National Bamboo Research Center, Hangzhou, 310012 Zhejiang Province China; 2 Chinese Academy of Forestry, Beijing, 100089 China

**Keywords:** Moso bamboo shoots, Rhizomes, Starch, Growth, AGPase, SBE, Starch metabolism-related genes

## Abstract

**Background:**

Both underground rhizomes/buds and above-ground Moso bamboo (*Phyllostachys heterocycla*) shoots/culms/branches are connected together into a close inter-connecting system in which nutrients are transported and shared among each organ. However, the starch storage and utilization mechanisms during bamboo shoot growth remain unclear. This study aimed to reveal in which organs starch was stored, how carbohydrates were transformed among each organ, and how the expression of key genes was regulated during bamboo shoot growth and developmental stages which should lay a foundation for developing new theoretical techniques for bamboo cultivation.

**Results:**

Based on changes of the NSC content, starch metabolism-related enzyme activity and gene expression from S0 to S3, we observed that starch grains were mainly elliptical in shape and proliferated through budding and constriction. Content of both soluble sugar and starch in bamboo shoot peaked at S0, in which the former decreased gradually, and the latter initially decreased and then increased as shoots grew. Starch synthesis-related enzymes (AGPase, GBSS and SBE) and starch hydrolase (α-amylase and β-amylase) activities exhibited the same dynamic change patterns as those of the starch content. From S0 to S3, the activity of starch synthesis-related enzyme and starch amylase in bamboo rhizome was significantly higher than that in bamboo shoot, while the NSC content in rhizomes was obviously lower than that in bamboo shoots. It was revealed by the comparative transcriptome analysis that the expression of starch synthesis-related enzyme-encoding genes were increased at S0, but reduced thereafter, with almost the same dynamic change tendency as the starch content and metabolism-related enzymes, especially during S0 and S1. It was revealed by the gene interaction analysis that AGPase and SBE were core genes for the starch and sucrose metabolism pathway.

**Conclusions:**

Bamboo shoots were the main organ in which starch was stored, while bamboo rhizome should be mainly functioned as a carbohydrate transportation channel and the second carbohydrate sink. Starch metabolism-related genes were expressed at the transcriptional level during underground growth, but at the post-transcriptional level during above-ground growth. It may be possible to enhance edible bamboo shoot quality for an alternative starch source through genetic engineering.

**Supplementary Information:**

The online version contains supplementary material available at 10.1186/s12870-021-03257-2.

## Background

Nutrients are usually stored in plant vegetative tissue mainly in the form of non-structural carbohydrates (NSCs), including soluble sugars and starch, which are important for plant growth and development [1–3]. Starch synthesis occurs in the following steps: starch synthesis substrates are catalysed by AGPase (adenosine diphosphate glucose pyrophosphorylase), amylose is synthesized by GBSS (granule-bound starch synthase), and amylopectin is synthesized by SS (soluble starch synthase) and SBE (Starch Branching Enzyme/Q enzyme) [4–6]. Transformation from starch to monosaccharides is triggered by starch hydrolases (α-amylase and β-amylase) as plants takes a relatively low soluble sugar content providing energy for growth and development [7, 8]. Starch accumulation in rice grains is positively correlated with the increase in AGPase and SSs [9]. At different developmental stages of wheat grains, the starch accumulation rate is significantly positively correlated with GBSS and SBE activities, and the accumulation process of amylopectin, amylose and the starch content after pollination follows a logistic growth equation [10]. At the molecular level, starch-related genes are involved in plant development. In rice, the expression of starch metabolism-encoding genes shows tissue and developmental period specificity. These genes are involved in the four main processes: construction of cellular machinery, embryonic development, starch synthesis in the embryo and kernel development [11]. The encoding wheat SBE gene controls starch synthesis at the transcriptome level, and encoding GBSS gene controls starch synthesis at the post-transcriptional level [10]. *AGPS1* (Adenosine diphosphate glucose pyrophosphorylase small subunit 1), *AGPL2* (Adenosine diphosphate glucose pyrophosphorylase lager subunit 2), *SSII* (Soluble starch synthase II), *SSIII* (Soluble starch synthase III), and *ISA* (Isoamylase) genes participate in starch metabolism in rice, wheat and potato [5, 11–18]. The starch (dry weight) content in the seeds of Moso bamboo (*Phyllostachys heterocycla*) is as high as 68.2%. The genes and starch properties of Moso bamboo are very similar to those of rice [19], but there are few studies on starch metabolism during the growth process of bamboo shoots.

Bamboo is known worldwide as one of the most important non-timber forest products thanks to their dual uses for culm timber and edible shoot moreover, bamboo is one of fastest growth plant species on earth and can grow at up to 1.2 m/day, and bamboo is one of most fascinating plant species because of their inter-connected system in which above-ground bamboo shoots/culms and underground rhizomes/buds are connected for transport and sharing of nutrients. Moso bamboo (*Phyllostachys heterocycla*), widely distributed in subtropical areas, and mainly found in China, covers a total area of 4.43 million hectares and is the most important bamboo species [20]. From late summer to early autumn, Moso bamboo lateral buds of underground mature rhizomes develop and grow into so-called winter shoots in winter, which are physiologically dormant. Winter shoots are broken in dormancy and start to grow from ground into so-called spring shoots under suitable temperature and moisture conditions. Spring shoots grow rapidly and can reach to 20 m height within 2 months [21, 22]. This species grows so very rapidly because of the cell division and elongation of a bottom-up meristem located at every shoot node [21, 23]. Stored NSCs also play an important role in the rapid growth of bamboo shoots, which have no photosynthesis ability; thus, energy is mainly derived from the degradation of sucrose [21, 24]. Song et al. [25] showed that assimilates from branches, leaves, culms and rhizomes of mature bamboo are transported to and provide energy for new spring shoots through underground rhizomes until they are self-sufficient in terms of photosynthesis. However, research on the utilization, storage and distribution of NSCs during the bamboo shoot growth process is limited and remains a critical and fundamental unknown. It is generally accepted that bamboo shoot NSCs are mainly stored in bamboo rhizomes, similar to such tuberous plant species as potato. However, such opinions are supported by little scientific evidences. Felisberto et al. [26] proposed that fresh young bamboo culms of *Dendrocalamus asper* can be used as a promising alternative starch source due to its rich starch composition being as high as 15%.

This research aimed to clarify the starch dynamic change pattern associated with the bamboo shoot growth process and identify the relationship between NSC storage and distribution and the growth and development of bamboo shoots by analysis of the spatial distribution and change of NSCs and starch contents, metabolic enzyme activity, and expression of key genes of bamboo shoots and connected rhizomes at four typical growth and developmental stages. The results provide a theoretical basis not only for guiding the efficient cultivation for bamboo shoots and culms, but also for creating a new use direction of bamboo shoots for a potential food and non-food starch industry since bamboo shoots have rich starch contents.

## Results

### Ultrastructural characteristics and distribution of starch grains in four growth and developmental stages of bamboo shoots and rhizomes

**Fig. 1:**
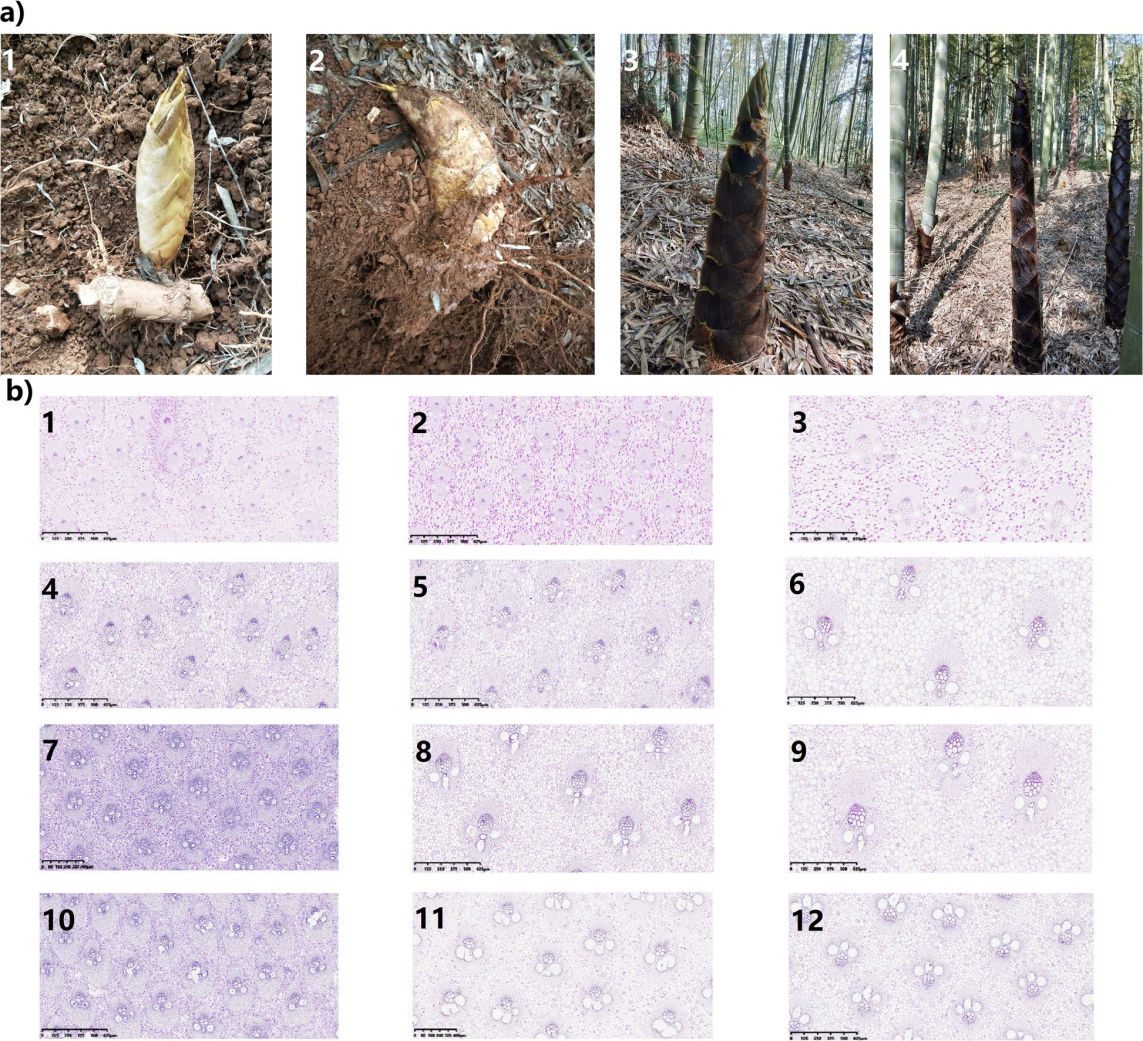
Tissue structure of bamboo shoots at different growth and developmental stages. **a** Four growth and developmental stages of Moso bamboo shoots (1, S0; 2, S1; 3, S3; 4, S4). **b** Tissue structure of bamboo shoots stained with PAS (× 4). 1, 4, 7, 10: the top (T) of bamboo shoots of S0, S1, S2, and S3, respectively. 2, 5, 8, 11: the middle (M) bamboo shoots of S0, S1, S2, and S3, respectively. 3, 6, 9, 12: the base (B) of bamboo shoots of S0, S1, S2, and S3, respectively

**Fig. 2:**
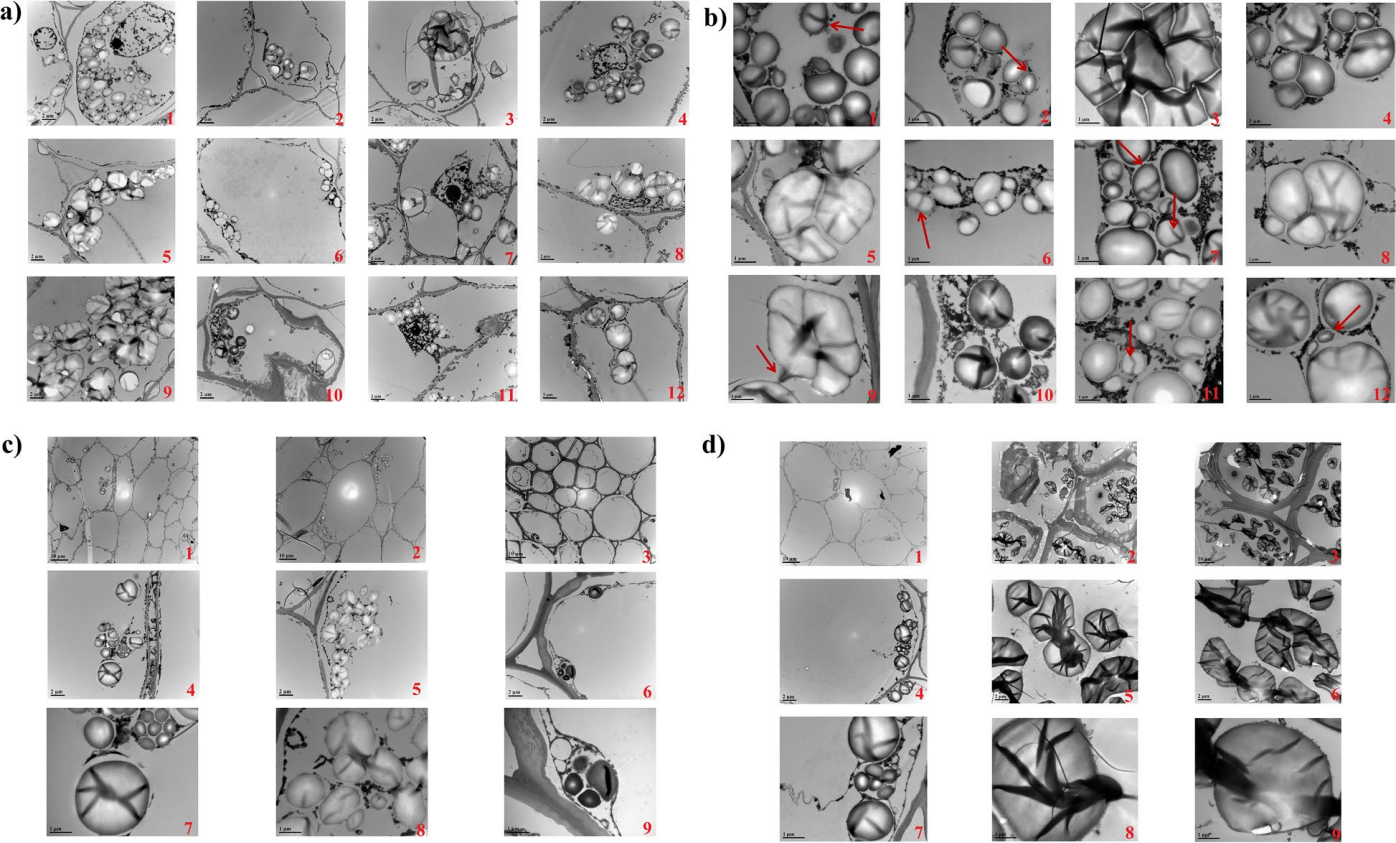
Microstructure analysis of starch in bamboo shoots at different growth and developmental stages. **a** Distribution of starch granules in the bamboo shoots via TEM (× 8000). The numbering in the upper left corner was the same as that in Fig. 1b. **b** Starch grain structure of bamboo shoots via TEM (× 25,000). the corresponds to the enlarged version, with arrows pointing to amyloplast proliferation. **c** Starch distribution of new buds on bamboo rhizomes via TEM. 1, 2, 3: new buds (× 2000) of one-year, two-year- and four-year-old rhizomes, respectively. 4, 5, 6: new buds of one-, two- and four-year-old rhizomes, respectively (× 8000). 7, 8, 9: new buds of one-, two- and four-year-old rhizomes, respectively (× 25,000). **d** Starch distribution of bamboo rhizomes via TEM. 1, 2, 3: One-r, two- and four-year-old bamboo rhizomes, respectively (× 2000). 4, 5, 6: One-, two- and four-year-old bamboo rhizomes, respectively (× 8000). 7, 8, 9: One-, two- and four-year-old bamboo rhizomes, respectively (× 25,000)

Li et al. [21] divided the growth process of Moso bamboo into 4 stages, i.e. the winter growth stage, early growth stage, late growth stage and after leaf expansion stage. In the early growth stage, bamboo shoots exhibit a slow-fast-slow growth pattern and grow up to 20 m for approximately 2 months [24, 25]. Based on bamboo shoot growth and development characteristics, bamboo shoots and rhizomes in the four different growth and developmental stages were chosen for this study: dormancy in winter (S0, underground dormant buds), up-earthing growth from underground in early spring (S1, winter shoots are broken in dormancy and are ready for emerging as spring shoots), rapid growth (S2, spring shoots are approximately 50 cm high aboveground) and slow growth (S3, spring shoots are approximately 150 cm high aboveground) (Fig. 1a). The results showed that, during S0, the starch granules of the top part of the bamboo shoots were small, the density was uniform, and the granules were distributed densely in an oval shape. The size of the base starch granules increased, the granules were extruded and formed into irregular arc-shaped polygons by each other, (Fig. 2a). The ultrastructural characteristics of the top starch granules in S1 and S2 were similar to those at S0. The morphology of starch grains at S3 was similar to that at S0, but there was no obvious polygon morphology after extrusion (Fig. 2a,b). In the four growth and developmental stages, the starch grains were mostly present in the form of compound grains and proliferated by budding and constriction (Fig. 2b). Starch grains were present in the parenchyma cells around the vascular bundles of the new buds in one-, two- and four-year-old rhizomes, which indicated that the buds were functioned in starch synthesis and accumulation after the formation of vascular bundles (Fig. 2d). Amorphous channels were observed in the starch grains of bamboo shoots and rhizomes at different stages via TEM (transmission electron microscope), especially in the parenchyma cells of two-- and four-year-old rhizomes. Amorphous channels play an important role in maintaining the crystal structure of starch grains and controlling the size of starch grains [27]. It is speculated that the starch structures of two- and four-year-old rhizomes are more stable than that of other rhizomes.

### Temporal and spatial distribution of NSCs in bamboo shoots and rhizomes

**Fig. 3:**
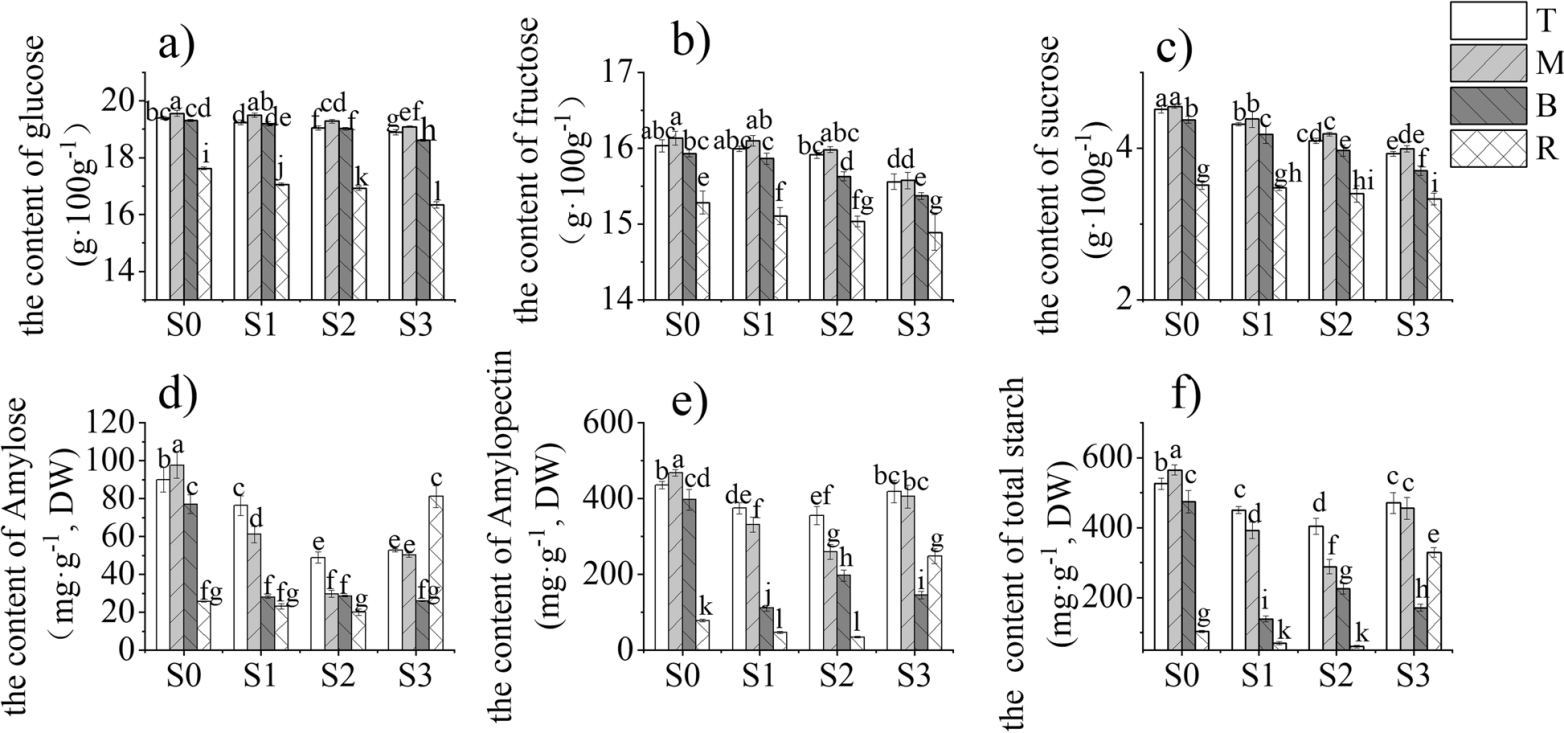
The contents of NSCs in different parts of Moso bamboo shoots at four growth and developmental stages. The different letters in each graph indicate significant differences at *p* < 0.05

The contents of sucrose, fructose, glucose and starch in the bamboo shoots were peaked at S0. As the bamboo shoots grew, these contents decreased (Fig. 3). From S0 to S3, the glucose content in the middle of shoot was the highest, followed by the top, the glucose content in the rhizome was the lowest. In addition, the glucose content of bamboo shoots decreased gradually with their growth and development, especially at S3, the glucose content of bamboo shoots and rhizomes decreased significantly, i.e., with the growth of bamboo shoots, the glucose content showed a decreasing trend, and the glucose content was inversely proportional to the growth of bamboo shoots (Fig. 3a). The dynamic change trend of fructose content was the same as that of glucose. With the increase of the height of bamboo shoots, the fructose content gradually decreased, and significantly decreased in S3. In the same stage, the fructose content change from high to low was in order: the middle part, top part, base part and bamboo rhizome (Fig. 3b). The change trend of sucrose content was similar to glucose and fructose. The content of sucrose decreased from S0 to S3, and the content of sucrose was the highest in the middle part of the bamboo shoots in each stage. At the same time, there is a similar phenomenon that the sucrose content of bamboo rhizomes is significantly lower than that of bamboo shoots (Fig. 3c). The contents of glucose, fructose and sucrose in different parts of bamboo shoots were consistent with different growth and development stages, but the contents of starch were different (Fig. 3f). In terms of space, the starch content in the middle part of was the highest at S0, followed by the top part. However, started from S1, the starch content in the top part was the highest, followed by the middle part and the bottom part. In terms of time, from S0 to S2, the content of each part of bamboo shoot decreased significantly, and at S3, the starch content showed an upward trend (Fig. 3f). The amylopectin content of bamboo shoots was 80–90%, and that of bamboo rhizome was 67–76% (Fig. 3e,f). The change trend of amylose and amylopectin and total starch is consistent as bamboo shoots grow and develop, amylose and amylopectin content was declined initially and risen later the amylose and amylopectin of basal part of bamboo shoot dropped significantly at S1 and S2 (Fig. 3d,e). It was worth noting that the content of NSC in bamboo rhizomes was significantly lower than that in bamboo shoots at different growth and developmental stages.

### Temporal and spatial changes of enzyme activities related to starch metabolism

**Fig. 4:**
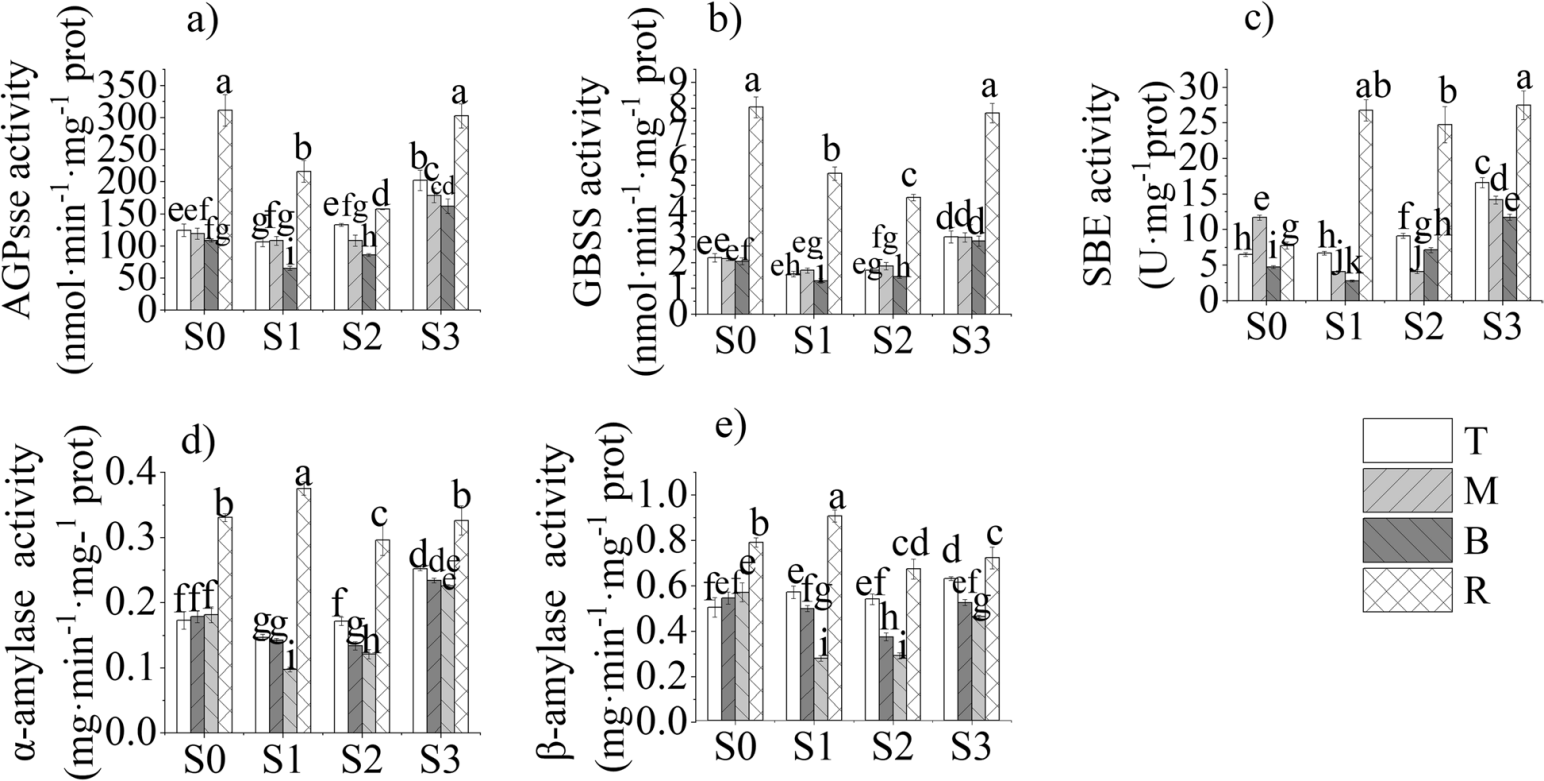
Dynamic changes in starch metabolism enzymes in Moso bamboo shoots and their connected rhizomes. **a** AGPase activity. **b** GBSS activity, **c** SBE activity. **d** α-Amylase activity. **e** β-Amylase activity. The different letters in each graph indicate significant differences at *p* < 0.05

This study revealed that the activities of starch synthesis-related enzymes in different parts of bamboo shoots at different stages shared similar patterns. The activity of AGPase and GBSS increased from the bottom to the top of bamboo shoots, but the activity of starch hydrolase increased from the top to the bottom at S0, and decreased gradually from the top to the bottom at the later stages (Fig. 4). From S0 to S2, the activities of starch synthesis-related enzymes such as AGPase and GBSS in the bamboo shoots decreased gradually but increased later at S3, which was in line with the variation of amylose and amylopectin contents (Figs. 3 and 4). The variations in AGPase, GBSS and SBE activities in the bamboo rhizomes were similar to those in the bamboo shoots. It was noted that the activities of all three kinds of enzymes in the rhizomes were significantly higher than those in the bamboo shoots.

The correlation analysis was conducted among starch metabolic enzyme activity, amylose and amylopectin content (Table 1). AGPase and SBE were significantly positively correlated with amylose content in the four growth and developmental stages. Amylase also had a significant positive correlation with amylose and amylopectin contents during S1-S3 stages. Therefore, AGPase, SBE and amylase were likely of great importance in the starch.synthesis and hydrolysis.

**Table 1:** Correlation analysis of starch metabolism enzymes and starch

	Amylose				Amylopectin			
	S0	S1	S2	S3	S0	S1	S2	S3
AGPase	0.675*	0.895**	0.884**	0.719**	0.536	0.934**	0.952**	0.686*
GBSS	0.068	0.749*	0.201	0.422	0.110	0.791*	0.421	0.408
SBE	0.794*	0.919**	0.751*	0.880**	0.841**	0.839**	0.484	0.850**
α-amylase	−0.275	0.959**	0.740*	0.779*	−0.310	0.973**	0.928**	0.735*
β-amylase	−0.398	0.984**	0.930**	0.821**	−0.418	0.975**	0.957**	0.779*

** significantly correlated at the *p* < 0.01 level

* Significant correlation at the *p* < 0.05 level

### Overview of gene expression in bamboo shoots at different growth and developmental stages

To investigate the expression of genes involved in starch metabolism from S0 to S3, the gene expression in the top and basal parts of bamboo shoots at different stages was measured through the Illumina sequencing platform. In total, 178.63 Gb of Clean Data were acquired. The Clean Data from each sample exceed 6.77 Gb. The percentage of Q30 bases was more than 95.86% (Table S1, Supplementary File 1), and the quality of RNA was fine. Sequence alignment was conducted between the Clean Data of the samples and the reference genome of Moso bamboo, and the mapping percentage ranged from 95.68 to 96.59% (Table S2, Supplementary File 1).

**Fig. 5:**
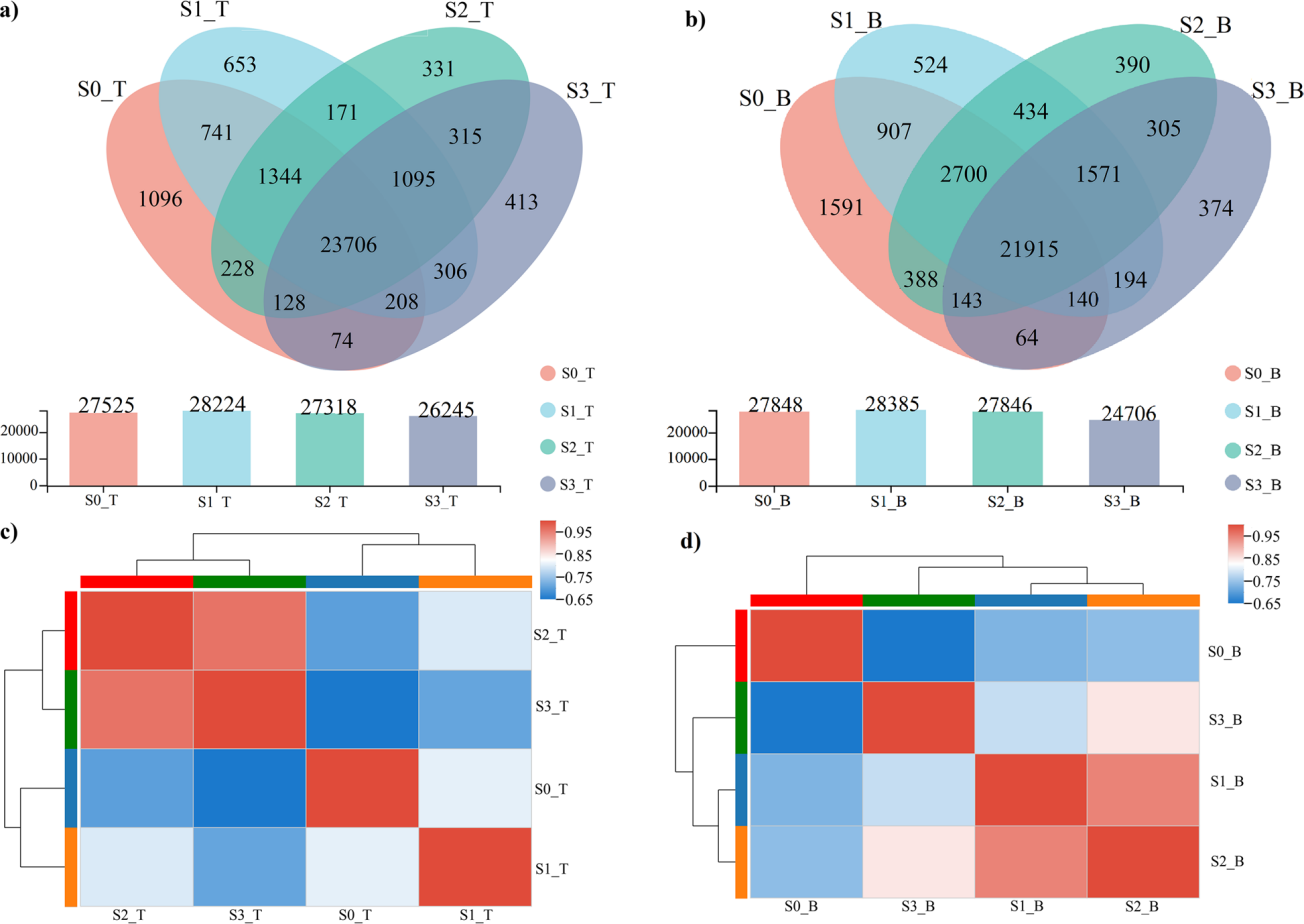
Overview of gene expression in Moso bamboo shoots at four growth and developmental stages. **a** Venn diagram of gene expression at the top of bamboo shoots at four growth and developmental stages. **b** Venn diagram of gene expression in the base of bamboo shoots at four growth and developmental stages. **c** Correlation heatmap of the top part of bamboo shoots at four growth and developmental stages. **d** Correlation heatmap of bamboo shoot basal parts at four growth and developmental stages. Each oval area of different colours in the Venn diagram represents the genes screened based on the expression level at the respective growth stage, and the data show the numbers of shared and specific genes at the different growth and developmental stages. The sum of all the numbers inside the oval represents the total number of genes at the respective growth stage. The numbers in intersecting regions show the number of shared genes at those stages. In the heatmaps, the right and lower sides represent bamboo shoots at different growth and developmental stages, while the left and upper sides represent sample clusters. The blocks of different colours indicate the correlations between any two growth and developmental stages

A total of 51,633 genes were detected in this analysis, including 42,001 known genes and 9632 unknown genes. There were 86,187 expressed transcripts, including 40,876 known transcripts and 45,311 unknown transcripts. Gene expression statistics of bamboo shoots at different stages were assessed by use of RSEM software at transcripts per million reads (TPM) as the expression index and selecting an expression level > =1. The amount of same gene expression levels in the top and basal parts of bamboo shoots at all four stages were 23,706 and 21,915, respectively (Fig. 5a,b). The gene expression at S3 was lower than that at the three other stages (Fig. 5a,b). Quantitative differential gene expression analysis of the bamboo shoots at different stages (DESeq2 software, screening threshold: |log2FC| > =1.000, padjust < 0.05) revealed differentially expressed genes (DEGs) of bamboo shoots at different stages (Table S3, Supplementary File 1). At S3, the numbers of DEGs in the top and basal parts of bamboo shoots was significantly higher than that at the other three stages, so there was apparently differences among the growth and developmental stages of the bamboo shoots. Correlation analysis of the samples at different stages (Fig. 5c, d) indicated that there were apparently two clusters in the top part at S0 and S1 and in the top part at S2 and S3. There were three clusters in the basal parts at S0, S1-S2 and S3, which corresponded to three periods: underground dormancy, fast growth after emerging out of the underground and slow growth. The clustering results verified the reliability of dividing bamboo shoot growth into different stages.

### Validation of RNA-Seq data by quantitative real-time PCR (qRT-PCR)

**Fig. 6:**
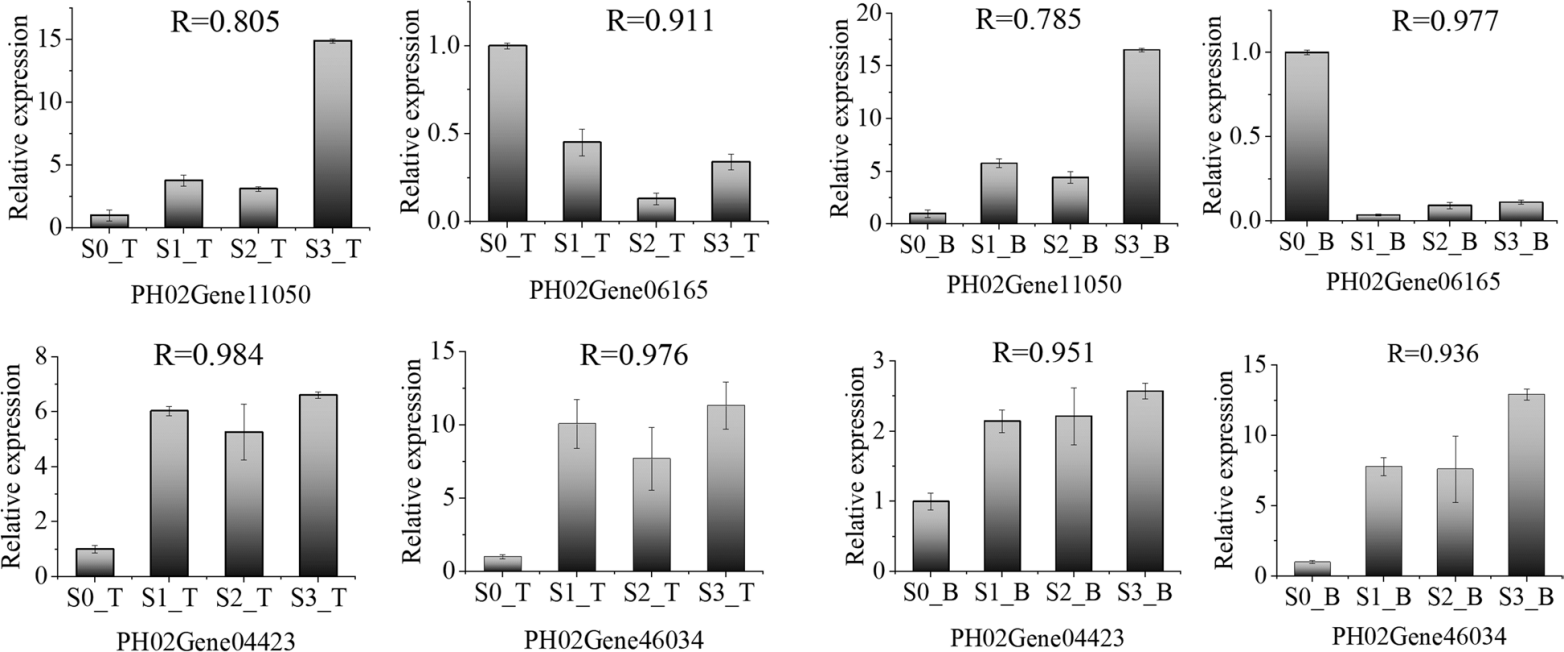
Verification of DEGs via qRT-PCR. The relative amount of mRNA (y-axis) is the ratio normalized to the amount of ACT (Actin) mRNA. The bamboo shoot growth and developmental stages are on shown on the x-axis. R indicates the correlation coefficient of the expression between the RNA-seq and qRT-PCR data (*p* < 0.05). The expression of each gene in S0 was set at 1.0

To validate the DEGs identified by RNA-seq, qRT-PCR was performed on four genes from the top and basal parts of Moso bamboo shoots at the four different growth and developmental stages. As expected, qRT-PCR confirmed that the expression trends were consistent with the DEG analysis via RNA-seq. The qRT-PCR data and transcriptome data were in a close agreement, which indicated that the transcriptome results were highly reliable. Pearson correlation coefficients of the bamboo shoot top parts ranged from 0.805 to 0.984, and those of the bamboo shoot basal parts ranged from 0.785 to 0.977 (Fig. 6).

### Differentially expressed genes involved in starch and sucrose metabolism pathways at different growth and developmental stages of bamboo shoots

To further study genes involved in bamboo shoot growth, GO functional annotation analysis of DEGs in the top and basal parts of bamboo shoots at different growth and developmental stages was conducted. The DEGs could be divided into three categories: those involved in biological processes, cellular components and molecular functions (Fig. S1, Supplementary File 2). Most DEGs involved in biological processes were related to cell processes and metabolic processes, most DEGs concerning cellular components were related to cellular organelles and cell components, and most DEGs involved in molecular functions were related to catalytic activities and linkage.

**Fig. 7:**
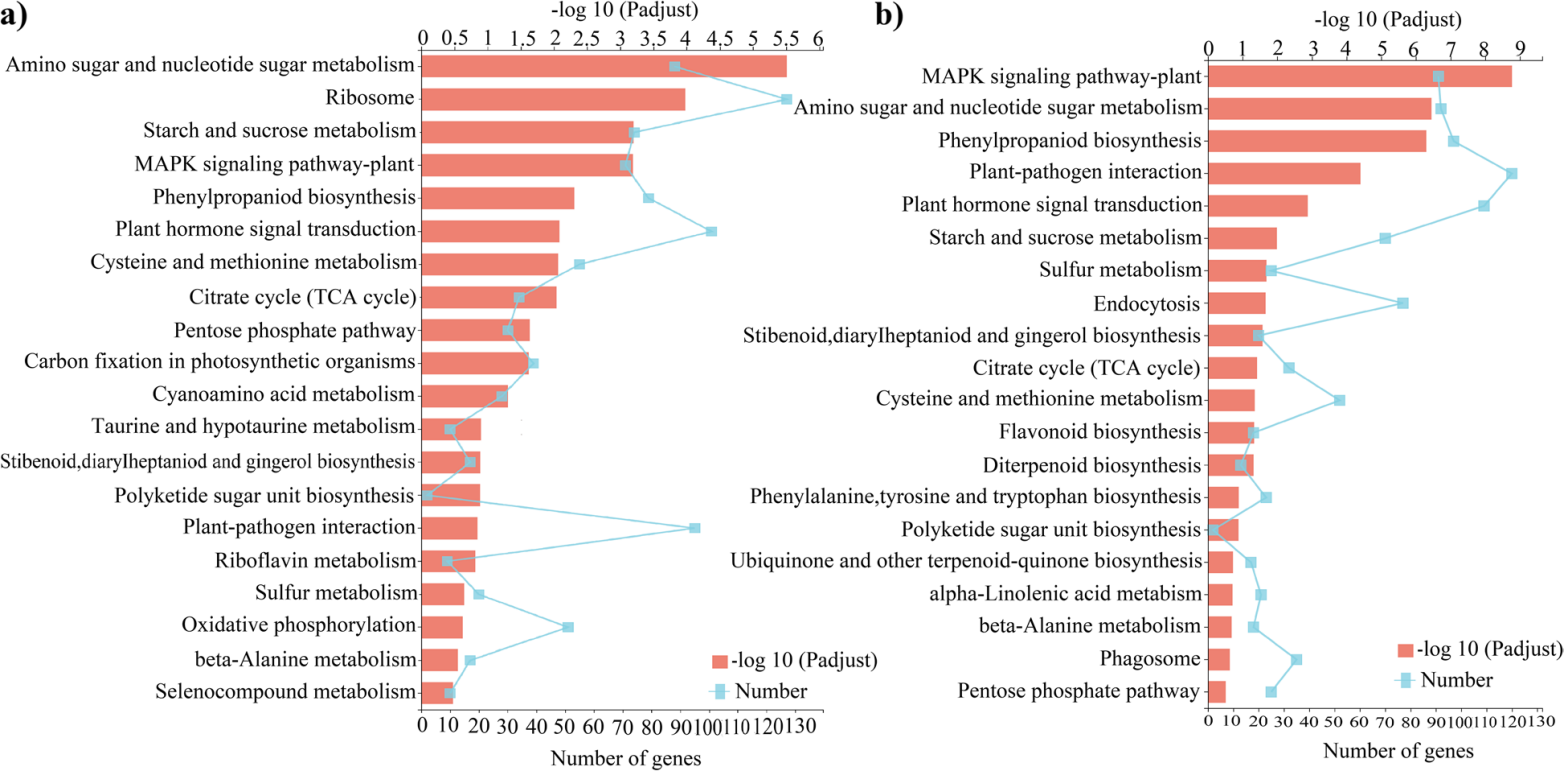
KEGG enrichment analysis of genes during Moso bamboo shoot growth and development **a** KEGG enrichment analysis of genes expressed in the top part of bamboo shoots. **b** KEGG enrichment analysis of genes expressed in the basal part of bamboo shoots. The vertical axis represents the KEGG pathways. The upper horizontal axis represents the number of genes aligned to a pathway, corresponding to the different points on the broken line. The lower horizontal axis represents the enrichment significance, corresponding to the lengths of the column. The lower the FDR is and the higher the -log10 (padjust) value is, the more significant the enrichment of the KEGG pathway

KEGG enrichment analysis showed that there were significant differences in the expression of genes related to the starch and sucrose metabolism pathways in the bamboo shoots at the four different growth and developmental stages (Fig. 7). When padjust< 0.5, the top 20 enrichment results indicated that 74 genes were involved in the starch and sucrose metabolism pathway in the top part of bamboo shoots and that 70 were involved in the starch and sucrose metabolism pathway in the basal part of bamboo shoots. Therefore, the starch and sucrose metabolism pathway is involved in bamboo shoot growth.

**Fig. 8:**
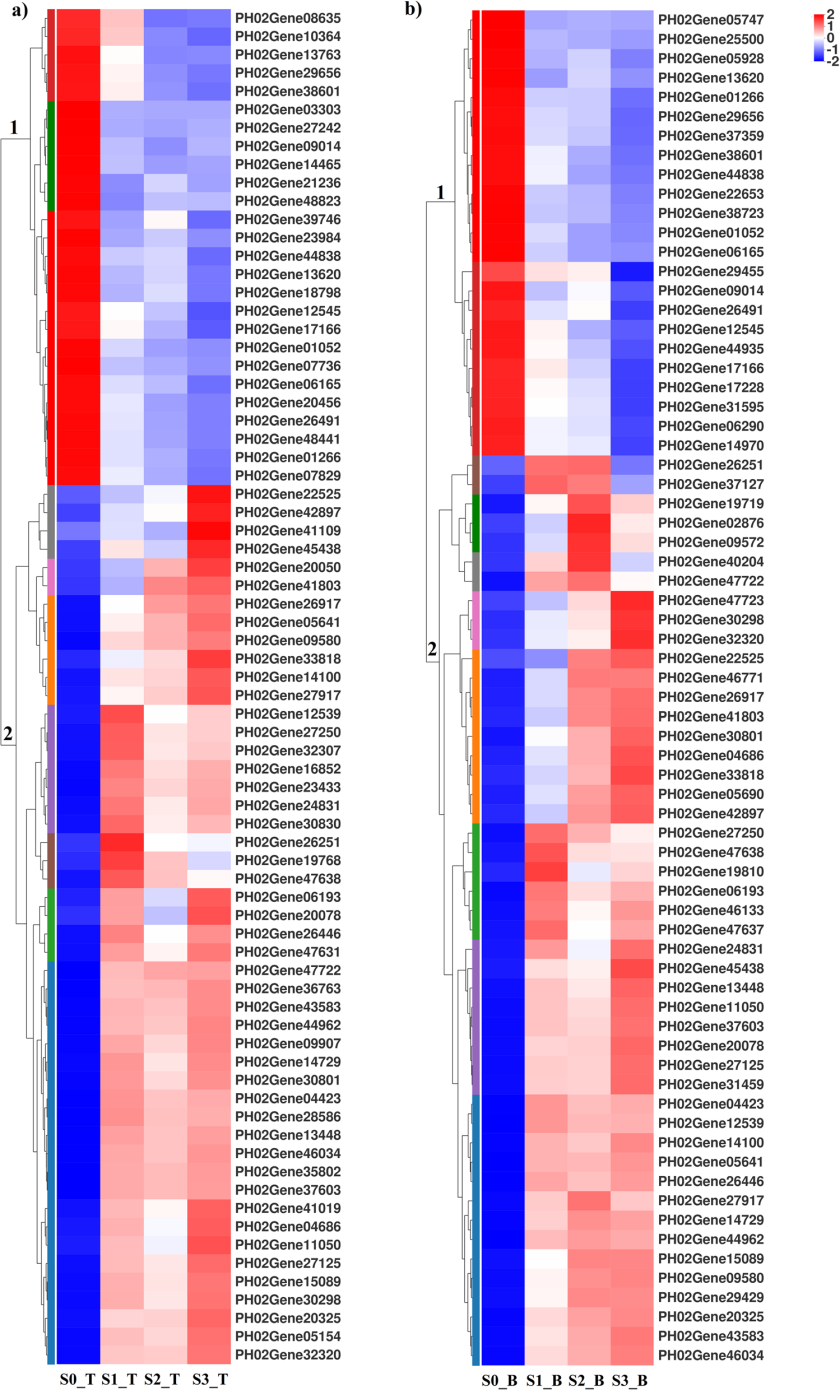
Expression of genes encoding starch and sucrose metabolism pathway in bamboo shoots at four growth and developmental stages. **a** Expression of genes involved in starch and sucrose metabolism pathway in the top part of bamboo shoots. **b** Expression of genes involved in starch and sucrose metabolism pathway in the basal part of bamboo shoots. Each column in the diagram represents a growth stage, and each row refers to a gene. The colours in the diagram show the expression values of each gene at every stage after the standardized process. The red colour indicates relatively high expression of the gene at that stage, while the blue colour indicates relatively low expression. The digits below the upper-right colour bar refer to the detailed variation tendency of expressed genes. On the left is the tree diagram of gene clusters and the block diagram of sub-clusters. On the right is the gene names. The closer two gene branches are, the more similar their expression levels

Cluster analysis was conducted for genes encoding the starch and sucrose metabolism pathway in the bamboo shoots at different growth and developmental stages (see Tables S4-S5 for detailed gene information, Supplementary File 1). There were two clusters of genes in the top and basal parts of bamboo shoots (Fig. 8). At S0, the expression of genes in cluster 1 reduced gradually as bamboo shoots grew, while the expression of genes in cluster 2 increased steadily during the same period. In general, among those involved in the starch and sucrose metabolism pathway, the genes encoding starch synthesis-related enzymes (e.g., PH02Gene12545, PH02Gene13602, PH02Gene44838 and PH02Gene06165) almost had the highest expression level in both the top and basal parts at S0 (Fig. 8).

### Function of genes encoding AGPase and SBE in bamboo shoots at different growth and developmental stages

**Fig. 9:**
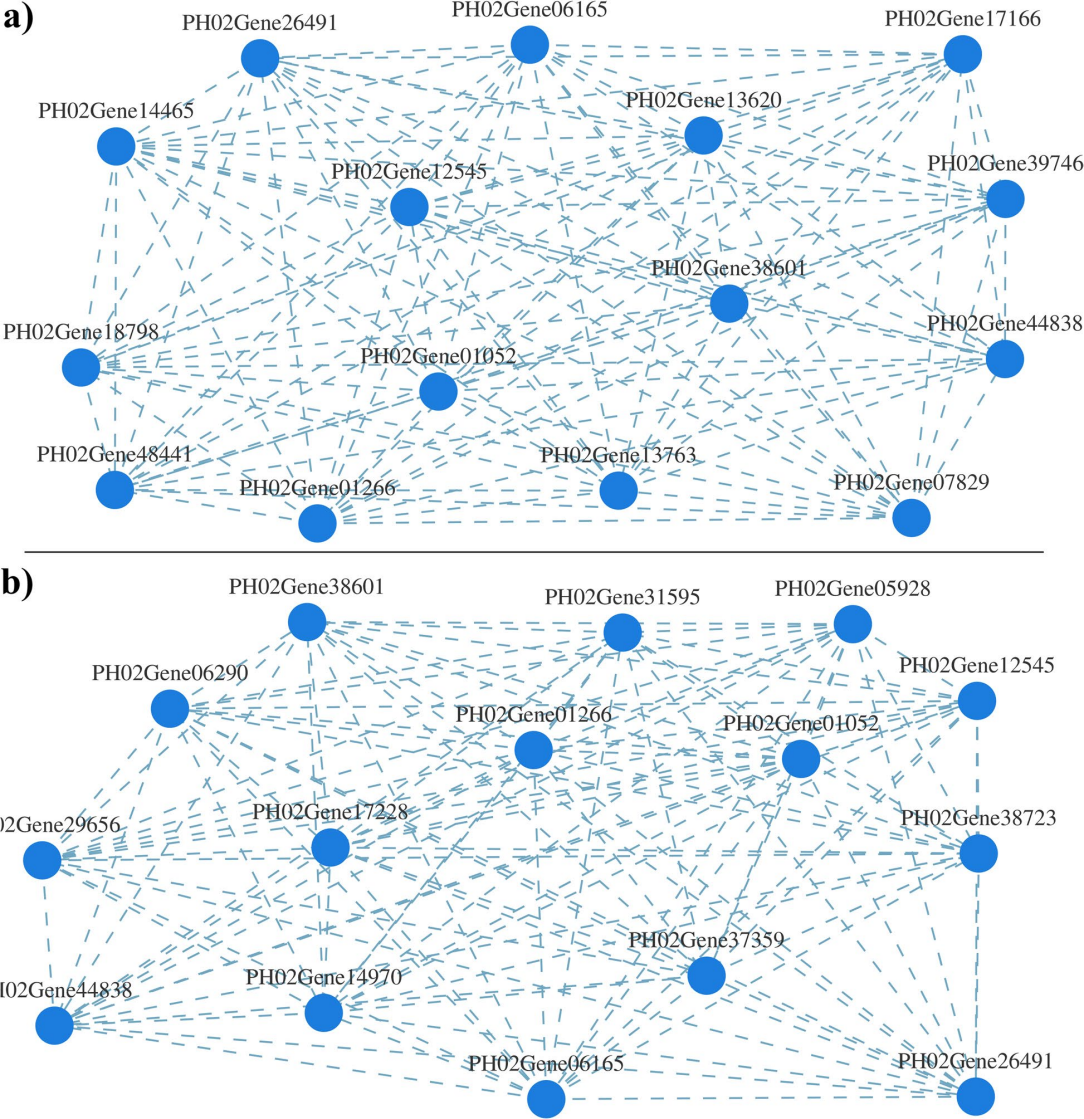
Gene co-expression network analysis. **a** Gene co-expression network analysis of the top part of bamboo shoots. **b** Gene co-expression network analysis of the basal part of bamboo shoots. Each node in the diagram represents a gene. Usually, the higher the node connectivity is (many nodes were connected to it), the more important the node

To analyse the interactions of encoding genes involved in starch and sucrose metabolism pathway, gene co-expression network analysis was conducted for genes involved in starch and sucrose metabolism in the top and basal parts of bamboo shoots (Fig. 9). Among the top fifteen most highly connected genes in the gene interaction network, seven in the top part of bamboo shoots were related to starch metabolism, and four of them encoded different AGPase subunits. The connectivity of these four genes was higher than that of other genes involved in starch and sucrose metabolism. Eleven genes in the basal part of bamboo shoots were related to starch metabolism, of which four genes encoded SBE and two encoded different AGPase subunits. Therefore, it was concluded that genes encoding AGPase and SBE have important functions in starch metabolism involved in growth and development processes in the top and basal parts of bamboo shoots respectively (Tables S6-S7, Supplementary File 1).

## Discussion

### The distribution of NSCs is related to the growth characteristics of bamboo shoots

Aboveground culms/shoots and underground rhizomes/buds of Moso bamboo is formed into a closed inter-connected system through which nutritional substances are transported and shared in each organ. Nutritional substances of the new shoot buds are supplied by the mother bamboo plant through rhizomes [25]. NSC is of great significance to the growth and development of plants. For example, NSC can maintain the survival of trees at low temperatures in high latitudes and high altitudes. NSC provides energy for vegetative growth of plants in Spring. NSC is stored in the stem for on-synchronous energy supply and demand during season stress [1, 2].

It is worth noting that starch grains were observed in parenchyma cells of bamboo shoots at different growth and developmental stages, especially in parenchyma cells surrounding vascular bundles. In addition, there were more NSC contents distributed in the upper part than in the base part (Fig. 3). The long-distance transport of plant organic matter is driven by water potential difference at both ends of the vascular bundle, the anatomical structure of bamboo determines that it mainly relies on longitudinal transport, and only transverse transport exists in nodes [28]. Therefore, it is more important for the growth and development of bamboo to carry out longitudinal transport of organic matter through vascular bundles. The starch and soluble sugars contents were peaked at S0, and the content was higher in the middle and top part than in the base part at all the four growth and developmental stages. There were two reasons for this. Firstly, On one hand, the water potential between the upper and lower parts of bamboo shoots was maintained, which drove organic matter transport from the bottom to the top and promoted the elongation of differentiated cells [29] . Deng et al. [29] studied the relationship between assimilate and water transport in *Fargesia yunnanensis*, they proposed that in longitudinal transport, assimilate transport was positively correlated with water pressure and potential. On another hand, in lateral transport, assimilates can be moved easily from the inner zone to the outer zone in response to water flow and sugar concentration gradients [29] . The water flow and sugar concentration gradients promoted the lateral transport of organic matter to supply growth and development. Besides, plasmodesmata are present between the phloem and storage cells during potato tuber formation, and assimilates are unloaded by the symplast pathway [30, 31]. In sink cells of barley, sugar is also transported by means of plasmodesmata [32]. The turgor pressure difference and transmembrane concentration gradient at both ends of plasmodesmata are important factors in determining symplastic transport [33]. Secondly, the growth and development of bamboo shoots began from the bottom part, cell division occurred robustly at the top, cell elongation was prominent in the middle, and cell wall thickening took place at the bottom. Cell wall thickening is the result of cellulose deposition, which is composed of glucose units linked by beta-1,4 linkages [24, 34, 35], so the contents of soluble sugars and starch at the bottom were lower than those at the middle and top. Therefore, we speculated that the content of NSC in the upper part of bamboo shoot was higher than that in the base, which was probably to maintain the water potential and facilitate the assimilate to be transported upward from the base, then completed the development of tissue structure. At the same time, starch stored around the vascular bundles, so as to meet the lateral transport concentration gradient and complete the growth and development of new shoots.

Soluble sugars (sucrose, fructose and glucose) function instantaneously for plant growth and development activities, such as respiration and the generation of osmotic pressure, while starch is stored as a substance for future use [36, 37]. In winter, bamboo shoots grow underground, they undergo structural differentiation, and thickening of the primary cell walls [38], all of which are relied on nutritional substances and energy supplies. Moreover, because the temperature is relatively low in winter, the content of sucrose is elevated in autumn and winter, which ensures the basic respiration of the plants and enhances their cold resistance [39]. Under suitable spring conditions, bamboo shoots break dormancy, emerge out of the underground and grow rapidly. The early fast growth stage primarily depends on cell division, and the energy for cell division is derived from sucrose degradation because bamboo shoots have almost no photosynthesis [21, 24]. Therefore, the gradual decrease of contents of glucose, fructose and sucrose in the bamboo shoots from S0 to S2 (Fig. 3) is associated with growth. Sucrose can be hydrolyzed to fructose and glucose, which are further converted into glucose-1-phosphate by several enzymes: hexokinase, phosphoglucose isomerase and phosphoglucomutase [40]. Transformation from starch to monosaccharides happens as plants have a relatively low soluble sugar content [7, 8]. Besides, when the content of soluble sugars was relatively low, the substrate for starch synthesis was at an insufficient level, and consequently, the starch content decreased (S0-S2). At S3, the starch content tended to increase, while the contents of soluble sugars were still relatively low. We speculated that this phenomenon may be resulted from slowing growth rate of bamboo shoots at S3, and the soluble sugar content needed for instantaneously function decreases, and the excess sugar is stored by starch, ready for the next fast growth. Therefore, the variance contents of glucose, fructose, sucrose and starch in the bamboo shoots was highly correlated with the shoot growth characteristics.

### Relationship among spatiotemporal changes in NSCs, enzymes involved in starch metabolism and the growth and development of bamboo shoots and rhizomes

When the soluble carbohydrate content in plants is relatively higher, sucrose is hydrolysed into fructose and glucose, which can be further converted to glucose-1-phosphate (the substrate for starch synthesis). Owing to the functions of AGPase, GBSS and SBE, this substrate is eventually converted to starch for storage [40]. When the soluble sugar content is relatively lower, starch is hydrolysed by α-amylase and β-amylase to soluble carbohydrates to fulfil the demand of plant growth and development [7, 8]. At S0, starch hydrolase activity enhanced (Fig. 4) to increase soluble sugar contents and ensure substance supply to thicken cell walls at the bottom of bamboo shoots (Figs. 1 and 2). From S1 to S3, the bottom part completed structural development (Figs. 1 and 2), while the top and middle parts are still undergoing vigorous cell division and elongation, which should require more energies than the basal part [21, 24]. Thus, hydrolase activity in these parts was higher than at the bottom. However, the soluble sugar contents in the middle and top part of bamboo shoots were higher than the baseal part, the starch synthase activity and starch content were also higher than the basal part, which could be resulted from the conversion of extra sugar to starch storage in the middle and top parts in order to maintain the difference in concentration between the upper and lower parts.by facilitating the transport of substances.

Moreover, the middle and top parts contained more soluble sugars, which indicated that the activity of starch synthesis-related enzymes was elevated and that starch synthesis increased, then starch content higher than bottom part.

In winter (S0), the bamboo shoots contained higher amounts of starch compared with those at the other stages(S1-S3) because bamboo shoots need to complete their underground growth and development at low temperature, and low temperature could facilitate starch accumulation and enhance the activities of enzymes involved in starch synthesis [41–43]. Moreover, AGPase is a rate-limited enzyme involved in starch synthesis [44]. Thus, the contents of amylose and amylopectin were higher in S0 than at the other stages. However, the activities of AGPase, GBSS and SBE at S3 were higher than those at S0, and the starch content was lower than that at S0. This should be related to the substrate of starch synthesis (sugar). In spring, bamboo shoots emerge out of the underground and grow quickly, which requires the involvement of sucrose [21, 24]. Sucrose and fructose are the substrates for starch synthesis [40]. With relatively few substrates for starch synthesis, low amounts of starch would accumulate. Starch content at S3 is higher than that in S1 and S2, because the growth rate of bamboo shoots at S3 was slower than in the previous stages, and the demand for soluble sugars decreased correspondingly [21]. And also due to starch synthesis-related enzyme activities were higher than S1 and S2 (Fig. 4). Extra sugars are converted to starch by sucrose synthase, AGPase, PGM, SBE, GBSS, SS,etc. and stored for future use [40].

It was noted that the activities of all three kinds of enzymes in rhizomes were significantly higher than those in shoots, but the contents of amylose and amylopectin in rhizomes were apparently lower than those in shoots (Figs. 3 and 4). This could be attributed to dynamic changes between starch synthesis and hydrolysis. Though the activities of starch synthesis-related enzymes in rhizomes, including AGPase, GBSS and SBE, were substantially higher, the activities of α-amylase and β-amylase in rhizomes were also notably higher than those in shoots, which indicated that starch hydrolysis was dominant in rhizomes. The variations in the activities of amylases in shoots were similar to those of starch synthesis-related enzymes. These activities were lower in shoots than in rhizomes, but the starch content was higher in shoots than in rhizomes. This indicated that starch synthesis was dominant in shoots.

### Starch are stored in the bamboo shoots rather than in the rhizomes

Presently, it is generally acknowledged that nutritional substances such as starch are mainly deposited in bamboo rhizomes for the growth and development of bamboo shoots and young bamboo culms. In this study, we proposes that bamboo shoots are the main organ for starch storage during their growth and development according to our findings: firstly, the contents of glucose, fructose, sucrose and starch in the bamboo shoots at all four stages were always significantly higher than those in the connected rhizomes (Fig. 3). Secondly, starch granules accumulated in newly-developed buds of one-,two-and four-year old rhizomes in autumn (Fig. 2c), which is totally in accordance with the findings which starch granules come up in the bamboo shoots as the vascular bundles were just formed [45]. It would be indicated that starch granules began to be deposited in the bamboo shoots when vascular tissues appeared. Thirdly, bamboo shoots mainly developed from the dynamically-developed rhizomes which were normally referred to the two- and three-year rhizomes. Multi-layer cell walls appeared in the fibre cells of bamboo two- and four-year-old rhizomes, and parenchyma cell walls thickened (Fig. 2d). Significant lignification of plants can hinder apoplastic transportation [29]. If starch is stored at rhizomes, it might take too long to transport starch-hydrolysed monosaccharides from parenchyma cells to vascular bundles. This could limit the transport speed of starch and assimilates stored at the rhizomes in a large and quick way which made it quiet difficult to meet the needs of nutrients and energy for fast growth of young bamboo shoots. In addition, according to the above discussion on the relationship between spatiotemporal changes in enzymes involved in starch metabolism and the growth and development of bamboo shoots and rhizomes, bamboo shoots mainly undergo starch synthesis, but bamboo rhiozmes do starch hydrolysis. Amylopectin in different parts of bamboo shoots at various stages was higher than bamboo rhizomes (Fig. 3). Increased amounts of amylopectin are beneficial to maintaining the structure of starch granules [46, 47], which further demonstrated that the starch structure in rhizomes is not as stable as that in the bamboo shoots, or rhizomes are prone to hydrolysis. Hence, we speculated that starch sinks were located mainly in the bamboo shoots rather than in the rhizomes, as commonly acknowledged.

It was concluded that bamboo rhizomes may be served as a transport channel of starch and soluble sugar between mother bamboo plants and new shoots before the new shoots can be functioned by photosynthesis and second carbohydrate sink for starch and soluble sugar storage for the growth and development of young shoots.

### Genes encoding starch metabolism-related enzymes are specific-stage expressed

As bamboo shoots grew, the expression of genes encoding starch synthesis-related enzymes reduced gradually. In contrast, the expression of genes encoding starch hydrolases (e.g., PH02Gene11050, PH02Gene33818 and PH02Gene32320) increased as the bamboo shoots grew, and was similar to that of the activity of starch metabolism-related enzymes in the bamboo shoots at the different stages (Fig. 4). However, at S3, the expression of genes encoding starch synthesis-related enzymes was significantly reduced (Fig. 8), starch synthesis-related enzyme activity was relatively high, and the starch content was increased compared to that at S2, was almost the same as that at S1 but was lower than that at S0. It could be induced by the below three reasons. First, transcript levels might not always be correlated with enzyme activity or protein levels but might be subject to post-transcriptional and post-translational modifications [10]. For example, the expression of genes encoding SBE is modified by transcriptional or post-transcriptional mechanisms, or both. Protein phosphorylation and protein-protein interactions might have an impact on starch synthesis and hydrolysis by altering SBE activity [48, 49]. Second, plant phenotypes are not only subject to genetic regulation but also susceptible to environmental conditions, which could alter starch contents by affecting sensitive starch synthases [50]. Third, the biological starch synthesis system requires coordinated expression of many genes in certain tissues at any specified developmental stage. Carbohydrate signals may also control starch synthesis [11, 51]. The expression of genes encoding sucrose synthetase and fructokinase, such as PH02Gene46034, PH02Gene27125 and PH02Gene04423, in the bamboo shoots was substantially increased at S3, which might facilitate starch synthesis. Therefore, regulating the expression of genes related to starch synthases was of great importance at the early growth and developmental stages of bamboo shoots especially at S0 and S1,. This is worthy of further research .

### The genes encoding AGPase and SBE were the critical genes involving in the growth and development of bamboo shoot

AGPase catalyses the conversion of glucose-1-phosphate and ATP to ADP-glucose. AGPase has a heterotetrameric structure composed of two large and two small subunits [52]. PH02Gene12545 and PH02Gene44838 encode AGPase large subunit 1 (AGPL1). PH02Gene17166 encodes AGPase large subunit 2 (AGPL2), and PH02Gene13620 encodes AGPase small subunit (AGPS). The expression of these genes encoding different AGPase subunits was significantly increased in the bamboo shoots at S0, especially in the top part, which was in accordance with AGPase activity variation. At S0, the starch content in the bamboo shoots was highest among all four growth and developmental stages. When the bamboo shoots grew, the expression of genes encoding subunits of AGPase was reduced gradually, AGPase activity tended to decrease, and starch content decreased (Figs. 3 and 4). Li et al. [53] overexpressed genes encoding subunits of AGPase in maize and found that every gene encoding the subunits of AGPase could enhance AGPase activity in maize; the combination of multiple genes had even better effects on enhancement of kernel weight and starch content.

Amylose biosynthesis is primarily attributed to genes encoding GBSS, while amylopectin biosynthesis is relied on coordinated interactions among at least 17 genes encoding proteins such as SS, SBE, ISA, PUL and PHO1 [12]. PH02Gene14970, PH02Gene31595 and PH02Gene06165 encode SBEI in bamboo shoots, while PH02Gene29656 encoded SBEIIB. At S0, their expression was significantly increased compared with that at S1 and S2, but at S3, their expression was reduced. Moreover, SBE activity at S3 increased notably, which was apparently not consistent with the related gene expression. Mutisya et al. [54] investigated the gene expression of SBEI and SBEIIB in the endosperm and embryonic cells of sorghum and found that these genes have similar gene expression profiles; however, the variation in SBE expression was not consistent with the variation in active SBE protein levels, and rhythmic interference of mRNA did not have a downstream impact on the accumulation of corresponding protein products. Satoh et al. [16] compared a group of plants expressing the rice SBEI-deficit gene with the control group of plants and found that the two groups of plants contained the same amount of starch; however, the fine structure of starch was altered, and the starting temperature for starch gelatinization was decreased. Therefore, it was determined here that the SBE gene was involved in regulating starch synthesis in the bamboo shoots at S0 and S1. However, at S2 and S3, post-transcriptional modification of the SBE gene may be required for its function. In summary, we proposed that starch metabolism-related genes played an important role at the transcriptional level during the underground growth and developmental stages (S0 and S1), while starch metabolism-related genes played an important role at the post-transcriptional level during the aboveground growth period (S2 and S3). During bamboo shoot growth, the genes encoding AGPase and SBE should be the critical genes involved in the starch and sucrose metabolism pathway.

Based on the results and discussions, we proposed a possible starch dynamic change scenario for the growth and developmental of Moso bamboo shoots: from late summer to early autumn, the lateral buds of rhizomes attached to mother bamboo plants received soluble sugars and other nutrients through rhizomes transported from surrounding mother bamboo plants and developed into winter shoots, starch synthesis-related genes were significantly expressed, starch synthesis-related enzyme activity increased, and the surplus soluble sugars were converted into starch and stored in the bamboo shoots for the subsequent rapid growth of the next spring shoots. In spring, the bamboo shoots broke dormancy and grew rapidly, which required a large amount of soluble sugars. The expression of starch synthesis-related genes in the bamboo shoots was reduced, and the expression of starch hydrolysis-related genes was increased. The corresponding activity of enzymes related to starch synthesis decreased, and starch hydrolase activity increased; thus, the starch content decreased, but starch was still accumulating. At S3, the growth rate slowed, the demand for soluble sugars decreased, and the starch synthase activity in the bamboo shoots increased again, during which the surplus soluble sugars were converted into starch in preparation for the next stage of rapid growth.

## Conclusion

It was concluded that starch supplied for the growth and development of bamboo shoots was mainly stored in the bamboo shoots at all four different growth and developmental stages or that the bamboo shoots that lacked the ability to photosynthesize behaved as the first carbohydrate sink for their growth and development. The main function of bamboo rhizome was the channel function between mother bamboo and new shoots, followed by the second carbohydrate sink. Starch metabolism predominantly involved starch synthesis or accumulation in the bamboo shoots but hydrolysis in the rhizomes. According to comparative transcriptomics, starch and sucrose metabolism pathway were involved in the growth and development of bamboo shoots. We concluded that starch and sucrose metabolism pathway genes likely exhibit growth and development-stage specificity. Starch metabolism-related genes played a role at the transcriptional level during the underground shoot growth period (S0 and S1), while starch metabolism-related genes played a role at the post-transcriptional level during the aboveground shoot growth period (S2 and S3). The AGPase (PH02Gene12545, PH02Gene44838, PH02Gene17166, PH02Gene13620) and SBE (PH02Gene31595, PH02Gene06165, PH02Gene29656) genes were the key genes involved in the starch and sucrose metabolism pathways during the different growth and developmental stages of bamboo shoots. It would be quite prospective to enhance edible bamboo shoot quality to have a proper balanced content of starch and soluble sugars, to cultivate large-diameter mature bamboo culms for improved use in the food industry, and to develop high-starch young culms as an alternative starch source through genetic engineering.

## Methods

### Plant materials and growth conditions

Bamboo shoots and rhizomes were obtained from commercial farms located in Wuxing district, Huzhou city, Zhejiang Province (WX, 30° 48′ N, 119° 59′ E). It has a northern subtropical climate with four distinct seasons, with rain and heat occurring in the same season. The annual average temperature is 15.8 °C, the annual average precipitation is approximately 1200 mm, and the frost-free period is 224–246 days. The sample collection complied with local regulations and did not require specific permission. From January to April 2020, winter bamboo shoots emerge from below ground and grow as spring shoots. Bamboo shoots and corresponding rhizomes were collected from plants at each of four stages: dormancy in winter (S0, underground dormant buds), upcoming growth from below ground in early spring (S1, winter shoots break dormancy and are ready for emerging as spring shoots), rapid growth (S2, spring shoots are approximately 50 cm high aboveground) and slow growth (S3, spring shoots are approximately 150 cm high aboveground). In each stage, three bamboo shoots of similar size and no mechanical damage were divided into top, middle and basal parts, marked as T, M and B, respectively. Its corresponding rhizome was labelled R. An approximately 1 cm^3^ sample was taken from the middle part of the bamboo shoot of each part and put into fixed solution (the lower part of the tissue was used when the top part was too brittle). The remaining bamboo shoots and rhizomes were transported to the laboratory and stored at − 80 °C after flash freezing in liquid nitrogen. These materials were used for the determination of glucose, fructose, sucrose, and starch contents and starch metabolism-related enzyme (AGPase, GBSS, SBE, α-amylase and β-amylase) activities. The tissue of top and basal part of Moso bamboo shoot also used for transcriptome sequencing. At the same time, 1 cm^3^ samples of one-, two- and four-year-old bamboo rhizomes and their corresponding lateral buds were taken and put into fixed solution for tissue structural observations. The plant material were stored in the laboratory of Dr. Xingcui Ding at China National Bamboo Research Center in China.

### Organizational structural observation and analysis

Optical microscope: After conventional paraffin embedding operations, the top, middle and basal samples were cut into transverse sections with a thickness of approximately 6 μm by a rotator slicer (RM2016, Shanghai Leica Instrument Co., Ltd., China). The sections were stained with PAS (periodic acid-Schiff) [55]. The stained sections were then observed and imaged with an optical microscope (Nikon Eclipse CI microscope, Nikon, Japan).

Electron microscope: Each sample was first fixed with 2.5% glutaraldehyde in phosphate buffer (0.1 M, pH 7.0). It was then subjected to double fixation, dehydration, infiltration, embedding, ultrathin sectioning and staining, and the starch granules were observed with a transmission electron microscope (TEM, Hitachi Model H-7650, Hitachi, Japan).

### Determination of soluble sugar and starch contents

Preparation of the solution to be tested: 0.5 g sample was added to 10 mL of 80% ethanol, extracted for 40 min at 80 °C and then centrifuged at 11,000×g for 5 min. The above procedure was repeated three times. The supernatants were combined, and the ethanol was evaporated in a boiling water bath. The residue was dissolved in 5 mL of ultrapure water and subsequently used to determine the contents of glucose, fructose and sucrose. The sucrose, fructose and glucose contents were determined via high-performance liquid chromatography (HPLC) [56]. The parameters used for determination were as follows: the injected volume was 5 μL, the analytical column (Waters Amide, 250 mm × 4,6 mm, i.d.,3.5 μm, Waters Corporation, USA) used for sugar (glucose, fructose and sucrose) analysis was equipped with an evaporative light scattering detector (ELSD 2000ES, Hangzhou Alltest Biotech Co.,Ltd., China), the mobile phase consisted of acetonitrile:water =70:30 (V/V), the flow rate was 1 mL·min^− 1^, the column temperature was 30 °C, the gas flow rate of the ELSD was 2 L·min^− 1^, the drift tube temperature was 85 °C, and the gain value was 2. The sugars in each sample were identified and quantified using a standard method, with commercial standards for used glucose, fructose and sucrose (Sigma, St. Louis, USA).

The amylose and amylopectin contents were measured by amylose and amylopectin reagent kits (ZDF-1-Y, ZHDF-1-Y, Suzhou Comin Biotechnology Co., Ltd., China). Starch extraction: 0.01 g sample was homogenized with 1 mL of ethanol solution. After fully homogenizing, the sample was extracted in a water bath at 80 °C for 30 min. Centrifugation was carried out at 25 °C for 3000 g for 5 min. The supernate was removed and 1 mL diethyl ether was added to precipitate for 5 min. Centrifugation was done again, then 1 mL KOH solution was added to the precipitate. The solution in water bath at 90 °C for 10 min then cooling. Adding 20 μL starch extract solution, 14 μLHCl solution, 120 μL distilled water, 2 μL KI-I_2_ solution, 44 μL distilled water solution successively. The amylose content was measure by the absorbance at 620 nm, while absorbance value was measured at 550 nm and 734 nm to determined amylopectin. The starch content was calculated as follows: starch content (mg·g^− 1^, DW) = amylose (mg·g^− 1^,DW) + amylopectin (mg·g^− 1^, DW).

### Starch-metabolizing enzyme activity measurement

The activity of starch synthase was determined by spectrophotometry. Crude extracts of starch synthase were prepared according to the methods of Nakamura et al. [57]. Adding enzyme extract [containing 10 ml of 100 mmol·L^− 1^ Tricine-NaOH (pH 7.5), 8 mmol·L^− 1^ MgCl_2_, 2 mmol·L^− 1^ EDTA, 50 mmol·L^− 1^ 2-mercaptoethanol, 12.5% (v/v) glycerol, and 5% (w/v) insoluble polyvinylpyrrolidone] to 1 g frozen sample, which was then homogenized with a pestle and mortar on ice. The homogenate was centrifuged at 10,000×g for 10 min at 4 °C. Next, the supernatant was kept at 2 °C, and then used as the crude enzyme solution (AGPase and SBE) to be measured. The above enzyme extract was added to the precipitate and used as the GBSS crude enzyme solution to be kept at 2 °C and measured. The activities of AGPase and GBSS of crude enzyme solution were measured according to the methods of Nakamura et al. [57] and Dai et al. [58]. Activity determination of SBE was performed according to the steps of Li et al. [59].

0.1 g sample was homogenized with 1 mL distilled water. The homogenate was extracted for 15 min at room temperature. The homogenate was shaken once every 5 min to make it fully extracted. Centrifuge for 10 min at 25 °C for 3000 g. adding distilled water to the supernatant, then it is crude amylase extract solution. α-amylase and β-amylase reagent kits (DFMA-1-Y and DFMB-1-Y, Suzhou Comin Biotechnology Co., Ltd., China) were used to determine the activities of amylase. The specific operation about α-amylase determined is as follows: 75 μL crude extract was taken, bathed in water at 70 °C for 15 min, then 75 μL sodium citrate solution (including soluble starch) was added after cooling. Then 150 μL 3, 5-dinitrosalicylic acid, NaOH, potassium sodium tartrate solution was added in water bath at 95 °C for 5 min. After cooling, the absorbance was measured at 540 nm. Distilled water was used as the control. β-amylase determined as follow: Amylase diluent was obtained by 1 mL of the crude amylase solution, adding 4 mL of distilled water and shaking it well for the determination of the total activity of (α + β) amylase. Seventy-five microliter amylase diluent, add 75 μL sodium citrate solution (including soluble starch), keep the temperature at 40 °C for 5 min, add 150 μL 3, 5-dinitrosalicylic acid, NaOH, potassium sodium tartrate solution, water bath at 95 °C for 5 min, cool down and measure the absorbance at 540 nm. The soluble protein determination method complied with the Bradford Coomassie brilliant blue method [60].

### Transcriptome sequencing and information analysis

Two gram samples of the top and basal tissues of bamboo shoots were taken, and total RNA was extracted by the TRIzol (Invitrogen, China) method. The concentration and purity of the RNA were then detected by a Nanodrop 2000 instrument(Thermo Fisher scientific, USA), the integrity of the RNA was detected via AGE (agarose gel electrophoresis), and the RIN value was determined by an Agilent 2100 instrument (Agilent, USA). An RNA library was constructed by a TruSeq™ RNA Sample Preparation Kit (Illumina, San Diego, CA). The library was based on the NovaSeq 6000 sequencing platform (Illumina,USA) for high-throughput sequencing. Sequencing was conducted by Shanghai Majorbio Bio-pharm Technology Co., Ltd., and the sequencing data were analysed on the free online platform of the Majorbio Cloud Platform (www.majorbio.com).

### qRT-PCR validation

To validate the transcriptome sequencing results, four DEGs were selected randomly and analysed via qRT-PCR. For qRT-PCR, total RNA extraction was performed as described above. qRT-PCR of bamboo shoots at four different growth and developmental stages was performed separately, with S0 used as the control. One microgram of total RNA was reverse-transcribed to first-stand cDNA using HiScript Q RT SuperMix for qPCR (+gDNA wiper) (Vazyme, Nanjing China). The specific primer pairs used for qRT-PCR were designed by Shanghai Majorbio Bio-pharm Technology Co., Ltd. (Table S8, Supplementary File 1). ACT (Actin) was used as an internal control [61]. The qRT-PCR primer sequences of ACT and four DEGs were shown in Table S8. PCR was conducted using an ABI 7500 fluorescence quantitative PCR instrument (Applied Biosystems, USA). The cycling conditions were 95 °C for the initial 5 min, followed by 40 cycles of 5 s at 95 °C, 30 s at 55 °C and 40 s at 72 °C. The experiments were repeated technically and biologically three times. RNA expression level were calculated using the 2^-△△Ct^ method [62]. Then Pearson correlation analysis of gene expression levels between qRT-PCR and RNA sequencing was carried out by SPSS, and Origin was used for plotting.

### Data analysis

One-way analysis of variance (ANOVA) (normal distribution) or Kruskal-Wallis test (abnormal distribution) was used by SPSS 21.0 software and Duncan post hoc test (*p* < 0.05) to compared the NSC content and starch metabolism-related enzyme activities different growth and developmental stages of Moso bamboo shoot at top, middle, bottom and rhizomes (three biological replicates). The results were expressed as the means ± standard deviations. Figures were constructed with Origin 2018 software, which ordinate is the mean of the three biological measurements, and the error bar is the standard deviation. Pearson’s correlation analysis (*p* < 0.05) was conducted among starch metabolic enzyme activity, amylose and amylopectin content of Moso bamboo shoot.

## Supplementary Information

**Additional file 1: Table S1** Sample sequencing data statistics. **Table S2** Statistics of sample sequencing comparisons. **Table S3** Expression of DEGs in different stages of Moso bamboo shoots. **Table S4** Heat map analysis of DEGs involved in starch and sucrose metabolism pathway at the top of Moso bamboo shoots. **Table S5** Heat map analysis of DEGs involved in starch and sucrose metabolism pathway at the base of Moso bamboo shoots. **Table S6** Co-expression network of DEGs at the top of Moso bamboo shoots. **Table S7** Co-expression network of DEGs at the base of Moso bamboo shoots. **Table S8** Sequences of qRT-PCR primers used in this study.

**Additional file 2: Figure S1** GO functional annotation analysis of DEGs at the top (left) and base (right) of Moso bamboo shoots.

## Data Availability

All raw sequencing datasets used in the study have been deposited in the NCBI database under BioProject PRJNA689987 (https://www.ncbi.nlm.nih.gov/Traces/study/?acc=PRJNA689987&o=acc_s%3Aa).

## Abbreviations

ACT: Actin
AGPase: Adenosine diphosphate glucose pyrophosphorylase
AGPL1: Adenosine diphosphate glucose pyrophosphorylase large subunit 1
AGPL2: Adenosine diphosphate glucose pyrophosphorylase large subunit 2
AGPS: Adenosine diphosphate glucose pyrophosphorylase small subunit
AGPS1: Adenosine diphosphate glucose pyrophosphorylase small subunit 1
ANOVA: Analysis of variance
B: Basal part of bamboo shoot
DW: Dry weight
DEG: Differentially expressed gene
ELSD: Evaporative light scattering detector
GBSS: Granule-bound starch synthase
HPLC: High-performance liquid chromatography
ISA: Isoamylase
KEGG: Kyoto Encyclopedia of Genes and Genomes
M: Middle part of bamboo shoot
Moso bamboo: *Phyllostachys heterocycla*
NSC: Non-structural carbohydrate
PAS: Periodic acid-Schiff
PGM: Phosphoglucomutase
PHO1: Phosphorylase 1
PUL: Pullulanase
qRT-PCR: Quantitative real-time polymerase chain reaction
R: Bamboo rhizome
SBE: Starch branching enzyme
SSs: Soluble starch synthase
SS II: Soluble starch synthase II
SS III: Soluble starch synthase III
T: Top part of bamboo shoot
TEM: Transmission electron microscope
TPM: Transcripts per million reads
