# Supplementary material for: Association among starch storage, metabolism, related genes and growth of Moso bamboo (Phyllostachys heterocycla) shoots
Source: BMC Plant Biol. 2021 Oct 20;21:477. doi: 10.1186/s12870-021-03257-2 (PMC8527747; doi:10.1186/s12870-021-03257-2)
Supplement: Supplementary file 1 — Additional file 1: Table S1 Sample sequencing data statistics. Table S2 Statistics of sample sequencing comparisons. Table S3 Expression of DEGs in different stages of Moso bamboo shoots. Table S4 Heat map analysis of DEGs involved in starch and sucrose metabolism pathway at the top of Moso bamboo shoots. Table S5 Heat map analysis of DEGs involved in starch and sucrose metabolism pathway at the base of Moso bamboo shoots. Table S6 Co-expression network of DEGs at the top of Moso bamboo shoots. Table S7 Co-expression network of DEGs at the base of Moso bamboo shoots. Table S8 Sequences of qRT-PCR primers used in this study. [file 12870_2021_3257_MOESM1_ESM.pdf]

Supplementary File 1

Table S1 Sample sequencing data statistics

Sample	Raw reads	Clean reads	Error rate(%)	Q20(%)	Q30(%)	GC content(%)
S0_T1	45790168	45407670	0.0233	98.68	95.86	52.49
S0_T2	47961938	47536530	0.0231	98.75	96.08	52.46
S0_T3	47953016	47546070	0.0233	98.71	95.96	52.69
S0_B1	47412630	46982928	0.0232	98.74	96.06	53.41
S0_B2	47152968	46728392	0.023	98.8	96.21	52.88
S0_B3	46082548	45688254	0.023	98.83	96.3	53.34
S1_T1	52051874	51640742	0.0232	98.73	96.03	54.55
S1_T2	51789682	51367640	0.023	98.82	96.29	54.37
S1_T3	49911624	49502850	0.0231	98.76	96.13	53.94
S1_B1	48014520	47622274	0.0228	98.88	96.52	55.48
S1_B2	56617666	56144040	0.023	98.78	96.25	55.12
S1_B3	52100624	51610414	0.0233	98.68	95.92	55.15
S2_T1	49891388	49475466	0.0229	98.84	96.35	54.01
S2_T2	49545084	49143304	0.0229	98.86	96.41	54.08
S2_T3	51848736	51438212	0.0228	98.91	96.52	54.66
S2_B1	52882350	52467454	0.0229	98.86	96.41	55.11
S2_B2	51963608	51534860	0.023	98.82	96.3	55.79
S2_B3	48934270	48524130	0.023	98.81	96.27	55.21
S3_T1	52737490	52324516	0.0231	98.76	96.12	54.55
S3_T2	56506580	56083198	0.0229	98.85	96.38	55.04
S3_T3	46930874	46454594	0.0229	98.86	96.4	54.37
S3_B1	52056954	51592478	0.023	98.8	96.29	55.83
S3_B2	53069056	52636282	0.023	98.79	96.24	56.26
S3_B3	51280482	50824242	0.0231	98.77	96.21	56.23

Note: (1)Sample:Name of Sample. (2)Raw reads: the total number of items in the original sequencing data (reads, representing a sequencing read, and one reads is one). (3)Clean reads: the total number of items in sequencing data after quality control. (4)Error rate(%): the average Error rate of sequencing bases corresponding to quality control data is generally less than 0.1%. (5)Q20(%), Q30(%): The quality of sequencing data after quality control is evaluated. Q20 and Q30 respectively refer to the percentage of bases with sequencing quality above 99% and 99.9% in the total base. Generally, Q20 is above 85% and Q30 is above 80%. (6)GC Content (%): The percentage of the sum of G and C bases corresponding to the quality control data in the total base.

Table S2 Statistics of sample sequencing comparisons

Sample	Total reads	Total mapped	Multiple mapped	Uniquely mapped
S0_T1	45407670	43620948(96.07%)	2599937(5.73%)	41021011(90.34%)
S0_T2	47536530	45698283(96.13%)	2499514(5.26%)	43198769(90.87%)
S0_T3	47546070	45704650(96.13%)	2825575(5.94%)	42879075(90.18%)
S0_B1	46982928	44955531(95.68%)	2900819(6.17%)	42054712(89.51%)
S0_B2	46728392	44792089(95.86%)	2411767(5.16%)	42380322(90.7%)
S0_B3	45688254	43809830(95.89%)	2640729(5.78%)	41169101(90.11%)
S1_T1	51640742	49630914(96.11%)	2995237(5.8%)	46635677(90.31%)
S1_T2	51367640	49399605(96.17%)	3747791(7.3%)	45651814(88.87%)
S1_T3	49502850	47566464(96.09%)	3642912(7.36%)	43923552(88.73%)
S1_B1	47622274	45669788(95.9%)	2192025(4.6%)	43477763(91.3%)
S1_B2	56144040	53734630(95.71%)	2606886(4.64%)	51127744(91.07%)
S1_B3	51610414	49499407(95.91%)	2954516(5.72%)	46544891(90.19%)
S2_T1	49475466	47619992(96.25%)	4061209(8.21%)	43558783(88.04%)
S2_T2	49143304	47275738(96.2%)	4525766(9.21%)	42749972(86.99%)
S2_T3	51438212	49572253(96.37%)	2590947(5.04%)	46981306(91.34%)
S2_B1	52467454	50586905(96.42%)	2870212(5.47%)	47716693(90.95%)
S2_B2	51534860	49543705(96.14%)	2784079(5.4%)	46759626(90.73%)
S2_B3	48524130	46592980(96.02%)	2337374(4.82%)	44255606(91.2%)
S3_T1	52324516	50371552(96.27%)	3665287(7.0%)	46706265(89.26%)
S3_T2	56083198	53915589(96.14%)	4236462(7.55%)	49679127(88.58%)
S3_T3	46454594	44868554(96.59%)	5097932(10.97%)	39770622(85.61%)
S3_B1	51592478	49409203(95.77%)	3665671(7.11%)	45743532(88.66%)
S3_B2	52636282	50635595(96.2%)	3060208(5.81%)	47575387(90.39%)
S3_B3	50824242	48934792(96.28%)	2604650(5.12%)	46330142(91.16%)

Note: (1)Sample: Name of Sample. (2)Total reads: the number of filtered sequences (i.e. Clean reads). (3)Total mapped: number of Clean reads that can be mapped into the genome. (4)Multiple mapped: number of Clean reads with Multiple mapped locations in the reference sequence. (5)Unique mapped: Number of Clean reads with a Unique alignment position in the reference sequence.

Table S3 Expression of DEGs in different stages of Moso bamboo shoots

Different stages	Total DEGs	Up DEGs	Down DEGs
S1_T	9633	5863	3770
S2_T	9749	5395	4354
S3_T	14388	7990	6398
S1_B	10470	6596	3874
S2_B	10771	6606	4165
S3_B	17852	9412	8440

Note: The differential gene expression numbers of S1_T, S2_T and S3_T were all compared with S0_T, and the differential gene expression numbers of S1_B, S2_B and S3_B were all compared with S0_B.

Table S4 Heat map analysis of DEGs involved in starch and sucrose metabolism pathway at the top of Moso bamboo shoots

Gene_id	Gene Description	S0_T	S1_T	S2_T	S3_T
PH02Gene01052	RecName: Full=Soluble starch synthase 2-2, chloroplastic/amyloplastic; AltName: Full=Soluble starch synthase II-2; Flags: Precursor	1.7030	-0.2784	-0.6506	-0.7740
PH02Gene01266	RecName: Full=Isoamylase 3, chloroplastic; Short=AtISA3; Flags: Precursor	1.6772	-0.2139	-0.5440	-0.9193
PH02Gene06165	RecName: Full=1,4-alpha-glucan-branching enzyme 1, chloroplastic/amyloplastic; AltName: Full=Starch branching enzyme I; Flags: Precursor	1.6682	-0.2360	-0.4557	-0.9766
PH02Gene07736	RecName: Full=Glucan endo-1,3-beta-glucosidase 4; AltName: Full=(1-)3)-beta-glucan endohydrolase 4; Short=(1-)3)-beta-glucanase 4; AltName: Full=Beta-1,3-endoglucan	1.7210	-0.4171	-0.5683	-0.7356
PH02Gene07829	RecName: Full=Probable sucrose-phosphate synthase 4; AltName: Full=Sucrose phosphate synthase 4F; Short=OsSPS4F; AltName: Full=UDP-glucose-fructose-phosphate gluco	1.6569	-0.1304	-0.5730	-0.9536
PH02Gene12545	RecName: Full=Glucose-1-phosphate adenylyltransferase large subunit 1, chloroplastic; AltName: Full=ADP-glucose pyrophosphorylase; AltName: Full=ADP-glucose syntha	1.5759	-0.0072	-0.4060	-1.1626
PH02Gene13620	RecName: Full=Glucose-1-phosphate adenylyltransferase small subunit, chloroplastic/amyloplastic; AltName: Full=ADP-glucose pyrophosphorylase; AltName: Full=ADP-glu	1.6866	-0.4760	-0.2921	-0.9185
PH02Gene17166	RecName: Full=Glucose-1-phosphate adenylyltransferase large subunit 2, chloroplastic; AltName: Full=ADP-glucose pyrophosphorylase; AltName: Full=ADP-glucose syntha	1.5785	0.0545	-0.5235	-1.1096
PH02Gene18798	RecName: Full=Probable alpha,alpha-trehalose-phosphate synthase	1.6816	-0.5221	-0.2425	-0.9170
PH02Gene20456	RecName: Full=Beta-fructofuranosidase, insoluble isoenzyme 4; AltName: Full=Cell wall beta-fructosidase 4; AltName: Full=Invertase 4; AltName: Full=OsCIN4; AltName:	1.6745	-0.1592	-0.6513	-0.8640
PH02Gene23984	RecName: Full=Probable alpha,alpha-trehalose-phosphate synthase	1.7134	-0.5854	-0.3576	-0.7704
PH02Gene26491	RecName: Full=Sucrose synthase 4; Short=OsSUS4; AltName: Full=Sucrose-UDP glucosyltransferase 4	1.6790	-0.1838	-0.6267	-0.8685
PH02Gene39746	RecName: Full=Probable inactive beta-glucosidase 14; Short=Os4bglu14; Flags: Precursor	1.6034	-0.6435	0.0466	-1.0064
PH02Gene44838	RecName: Full=Glucose-1-phosphate adenylyltransferase large subunit 1,	1.6609	-0.3617	-0.2843	-1.0148

	chloroplastic/amyloplastic; AltName: Full=ADP-glucose pyrophosphorylase; AltName: Full=ADP-gl				
PH02Gene48441	RecName: Full=Sucrose synthase 4; Short=OsSUS4; AltName: Full=Sucrose-UDP glucosyltransferase 4	1.6844	-0.2068	-0.6198	-0.8579
PH02Gene03303	RecName: Full=Beta-glucosidase 12; Short=Os4bglu12; Flags: Precursor	1.7318	-0.5503	-0.5907	-0.5907
PH02Gene09014	RecName: Full=Beta-glucosidase 25; Short=Os6bglu25; Flags: Precursor	1.7189	-0.4450	-0.7712	-0.5027
PH02Gene14465	RecName: Full=Nudix hydrolase 14, chloroplastic; Short=AtNUDT14; AltName: Full=ADP-sugar diphosphatase; Short=AtASPP; Flags: Precursor	1.7249	-0.4304	-0.6744	-0.6202
PH02Gene21236	RecName: Full=Probable alpha,alpha-trehalose-phosphate synthase	1.7037	-0.7810	-0.2858	-0.6369
PH02Gene27242	RecName: Full=Glucose-1-phosphate adenylyltransferase large subunit 1, chloroplastic; AltName: Full=ADP-glucose pyrophosphorylase; AltName: Full=ADP-glucose syntha	1.7311	-0.5667	-0.6278	-0.5366
PH02Gene48823	RecName: Full=Alpha,alpha-trehalose-phosphate synthase	1.7116	-0.8202	-0.4307	-0.4607
PH02Gene04423	RecName: Full=Sucrose synthase 1; Short=OsSUS1; AltName: Full=Sucrose synthase 2; Short=RSs2; AltName: Full=Sucrose-UDP glucosyltransferase 1	-1.7206	0.7370	0.4121	0.5715
PH02Gene04686	RecName: Full=Beta-glucosidase 31; Short=Os9bglu31; Flags: Precursor	-1.5823	0.5269	-0.0475	1.1029
PH02Gene05154	RecName: Full=Endoglucanase 23; AltName: Full=Endo-1,4-beta glucanase 23; AltName: Full=OsGLU12; Flags: Precursor	-1.6774	0.4422	0.2802	0.9551
PH02Gene09907	RecName: Full=Phosphoglucomutase, cytoplasmic 2; Short=PGM 2; AltName: Full=Glucose phosphomutase 2	-1.7001	0.5992	0.2817	0.8193
PH02Gene11050	RecName: Full=Beta-amylase 1, chloroplastic; AltName: Full=1,4-alpha-D-glucan maltohydrolase; AltName: Full=Beta-amylase 7; AltName: Full=Thioredoxin-regulated beta	-1.5489	0.4611	-0.0872	1.1750
PH02Gene13448	RecName: Full=Glucose-6-phosphate isomerase, cytosolic; Short=GPI; AltName: Full=Phosphoglucose isomerase; Short=PGI; AltName: Full=Phosphohexose isomerase; Short=PH	-1.7234	0.6507	0.4118	0.6610
PH02Gene14729	RecName: Full=Probable alpha,alpha-trehalose-phosphate synthase	-1.6827	0.7143	0.1766	0.7918
PH02Gene15089	RecName: Full=Probable alpha,alpha-trehalose-phosphate synthase	-1.6714	0.5447	0.1925	0.9342

PH02Gene20325	RecName: Full=Probable trehalose-phosphate phosphatase 1; Short=OsTPP1; AltName: Full=Trehalose 6-phosphate phosphatase	-1.6522	0.2872	0.3246	1.0404
PH02Gene27125	RecName: Full=Fructokinase-1; AltName: Full=Fructokinase I; AltName: Full=OsFKI	-1.6510	0.4712	0.1678	1.0120
PH02Gene28586	RecName: Full=Glucan endo-1,3-beta-glucosidase 6; AltName: Full=(1-)3)-beta-glucan endohydrolase 6; Short=(1-)3)-beta-glucanase 6; AltName: Full=Beta-1,3-endoglucan	-1.7207	0.7469	0.4275	0.5463
PH02Gene30298	RecName: Full=UTP--glucose-1-phosphate uridylyltransferase; AltName: Full=UDP-glucose pyrophosphorylase; Short=UDPGP; Short=UGPase	-1.6661	0.5480	0.1725	0.9456
PH02Gene30801	RecName: Full=Alpha,alpha-trehalose-phosphate synthase	-1.7013	0.6926	0.2622	0.7465
PH02Gene32320	RecName: Full=Beta-amylase 3, chloroplastic; AltName: Full=1,4-alpha-D-glucan maltohydrolase; AltName: Full=Beta-amylase 8; AltName: Full=Chloroplast beta-amylase;	-1.6837	0.3910	0.3489	0.9437
PH02Gene35802	RecName: Full=Phosphoglucomutase, cytoplasmic 2; Short=PGM 2; AltName: Full=Glucose phosphomutase 2	-1.7269	0.5750	0.4675	0.6845
PH02Gene36763	RecName: Full=Lysosomal beta glucosidase; Flags: Precursor	-1.7173	0.4449	0.4890	0.7834
PH02Gene37603	RecName: Full=Fructokinase-2; AltName: Full=Fructokinase II; AltName: Full=OsFKII	-1.7278	0.5824	0.4735	0.6719
PH02Gene41019	RecName: Full=Hexokinase-3; AltName: Full=Hexokinase-8	-1.6151	0.4821	0.0579	1.0751
PH02Gene43583	RecName: Full=Fructokinase-2; AltName: Full=Fructokinase II; AltName: Full=OsFKII	-1.7149	0.5048	0.4152	0.7949
PH02Gene44962	RecName: Full=Beta-glucosidase 25; Short=Os6bglu25; Flags: Precursor	-1.7094	0.4883	0.3940	0.8271
PH02Gene46034	RecName: Full=Sucrose synthase 1; Short=OsSUS1; AltName: Full=Sucrose synthase 2; Short=RSs2; AltName: Full=Sucrose-UDP glucosyltransferase 1	-1.7212	0.5877	0.4090	0.7245
PH02Gene47722	RecName: Full=Sucrose synthase 7; Short=OsSUS7; AltName: Full=Sucrose-UDP glucosyltransferase 7	-1.7280	0.4672	0.6064	0.6544
PH02Gene05641	RecName: Full=UTP--glucose-1-phosphate uridylyltransferase; AltName: Full=UDP-glucose pyrophosphorylase; Short=UDPGP; Short=UGPase	-1.6404	0.1072	0.5194	1.0138
PH02Gene09580	RecName: Full=Sucrose:sucrose 1-fructosyltransferase; AltName: Full=Sucrose 1(F)-fructosyltransferase; AltName: Full=Sucrose:sucrose 1(F)-beta-D-fructosyltransferase	-1.6889	0.2671	0.5300	0.8918
PH02Gene14100	RecName: Full=Lysosomal beta glucosidase; Flags: Precursor	-1.6140	0.1884	0.2985	1.1272

PH02Gene26917	RecName: Full=Probable trehalose-phosphate phosphatase 7; Short=OsTPP7; AltName: Full=Trehalose 6-phosphate phosphatase	-1.6302	0.0108	0.6848	0.9346
PH02Gene27917	RecName: Full=Sucrose synthase 7; Short=OsSUS7; AltName: Full=Sucrose-UDP glucosyltransferase 7	-1.5873	0.0725	0.3525	1.1623
PH02Gene33818	RecName: Full=Beta-amylase 1, chloroplastic; AltName: Full=1,4-alpha-D-glucan maltohydrolase; AltName: Full=Beta-amylase 7; AltName: Full=Thioredoxin-regulated beta	-1.4746	-0.1084	0.2619	1.3211
PH02Gene06193	RecName: Full=Beta-fructofuranosidase, insoluble isoenzyme 2; AltName: Full=Cell wall beta-fructosidase 2; AltName: Full=Invertase 2; AltName: Full=OsCIN2; AltName	-1.5056	0.6623	-0.2635	1.1068
PH02Gene20078	RecName: Full=Sucrose:sucrose 1-fructosyltransferase; AltName: Full=Sucrose 1(F)-fructosyltransferase; AltName: Full=Sucrose:sucrose 1(F)-beta-D-fructosyltransferase	-1.4129	0.6516	-0.4222	1.1835
PH02Gene26446	RecName: Full=Beta-fructofuranosidase, insoluble isoenzyme 1; AltName: Full=Cell wall beta-fructosidase 1; AltName: Full=Invertase 1; AltName: Full=OsCIN1; AltName	-1.6366	0.8507	0.0128	0.7731
PH02Gene47631	RecName: Full=Endoglucanase 23; AltName: Full=Endo-1,4-beta glucanase 23; AltName: Full=OsGLU12; Flags: Precursor	-1.6516	0.6660	0.0787	0.9069
PH02Gene08635	RecName: Full=Beta-glucosidase 31; Short=Os9bglu31; Flags: Precursor	1.4520	0.4059	-0.9532	-0.9046
PH02Gene10364	RecName: Full=Probable trehalase; AltName: Full=Alpha,alpha-trehalase; AltName: Full=Alpha,alpha-trehalose glucohydrolase	1.4685	0.3676	-0.8106	-1.0254
PH02Gene13763	RecName: Full=Alpha,alpha-trehalose-phosphate synthase	1.6269	0.0180	-0.8301	-0.8147
PH02Gene29656	RecName: Full=1,4-alpha-glucan-branching enzyme 2, chloroplastic/amyloplastic; AltName: Full=Q-enzyme; AltName: Full=Starch-branching enzyme IIB; Flags: Precursor	1.6028	0.0787	-0.7662	-0.9153
PH02Gene38601	RecName: Full=Beta-glucosidase A; AltName: Full=Beta-D-glucoside glucohydrolase A; AltName: Full=Cellobiase A; AltName: Full=Gentiobiase A; Flags: Precursor	1.5863	0.1130	-0.7326	-0.9666
PH02Gene12539	RecName: Full=Endoglucanase 10; AltName: Full=Endo-1,4-beta glucanase 10; AltName: Full=OsGLU2	-1.5594	1.2051	0.0141	0.3401
PH02Gene16852	RecName: Full=Beta-fructofuranosidase, insoluble isoenzyme 2; AltName: Full=Cell wall beta-fructosidase 2; AltName: Full=Invertase 2; AltName: Full=OsCIN2; AltName	-1.6811	0.9078	0.2273	0.5460

PH02Gene23433	RecName: Full=Beta-glucosidase 4; Short=Os1bglu4	-1.7001	0.8317	0.2915	0.5769
PH02Gene24831	RecName: Full=Hexokinase-5; AltName: Full=Hexokinase I	-1.6639	0.9214	0.1402	0.6023
PH02Gene27250	RecName: Full=Endoglucanase 10; AltName: Full=Endo-1,4-beta glucanase 10; AltName: Full=OsGLU2	-1.6231	1.1034	0.1787	0.3410
PH02Gene30830	RecName: Full=Lysosomal beta glucosidase; Flags: Precursor	-1.6386	1.0310	0.1202	0.4875
PH02Gene32307	RecName: Full=Probable alpha-glucosidase Os06g0675700; AltName: Full=Maltase; Flags: Precursor	-1.6250	1.0945	0.1635	0.3670
PH02Gene19768	RecName: Full=Alpha-amylase type A isozyme; AltName: Full=1,4-alpha-D-glucan glucanohydrolase; AltName: Full=AMY1; AltName: Full=Low pI alpha-amylase; Flags: Precurso	-1.4410	1.2971	0.4115	-0.2676
PH02Gene26251	RecName: Full=Probable trehalase; AltName: Full=Alpha,alpha-trehalase; AltName: Full=Alpha,alpha-trehalose glucohydrolase	-1.3779	1.4479	0.0014	-0.0714
PH02Gene47638	RecName: Full=Beta-glucosidase 28; Short=Os8bglu28; Flags: Precursor	-1.5953	1.1301	0.4192	0.0460
PH02Gene20050	RecName: Full=Beta-glucosidase 34; Short=Os10bglu34; Flags: Precursor	-1.3525	-0.4644	0.5183	1.2986
PH02Gene41803	RecName: Full=Beta-glucosidase 16; Short=Os4bglu16; Flags: Precursor	-1.3692	-0.5329	0.8244	1.0778
PH02Gene22525	RecName: Full=Probable alpha,alpha-trehalose-phosphate synthase	-1.1097	-0.4318	-0.0641	1.6056
PH02Gene41109	RecName: Full=Probable trehalose-phosphate phosphatase 6; Short=OsTPP6; AltName: Full=Trehalose 6-phosphate phosphatase	-0.9198	-0.2185	-0.5396	1.6778
PH02Gene42897	RecName: Full=Probable alpha,alpha-trehalose-phosphate synthase	-1.2927	-0.2320	0.0164	1.5083
PH02Gene45438	RecName: Full=Hexokinase-7; AltName: Full=Hexokinase-6	-1.3082	0.1779	-0.3344	1.4647

Note:(1)description: name of gene code. (2)There are four growth and development stages of bamboo shoots: the expression level after standardization.

Table S5 Heat map analysis of DEGs involved in starch and sucrose metabolism pathway at the base of Moso bamboo shoots

Gene_id	Gene Description	S0_B	S1_B	S2_B	S3_B
PH02Gene01052	RecName: Full=Soluble starch synthase 2-2, chloroplastic/amyloplastic; AltName: Full=Soluble starch synthase II-2; Flags: Precursor	1.6965	-0.2487	-0.6461	-0.8017
PH02Gene01266	RecName: Full=Isoamylase 3, chloroplastic; Short=AtISA3; Flags: Precursor	1.6725	-0.3381	-0.3525	-0.9820
PH02Gene05747	RecName: Full=Probable sucrose-phosphate synthase 1; AltName: Full=UDP-glucose-fructose-phosphate glucosyltransferase	1.7316	-0.5963	-0.5390	-0.5963
PH02Gene05928	RecName: Full=Isoamylase 3, chloroplastic; Short=AtISA3; Flags: Precursor	1.6990	-0.5285	-0.3120	-0.8584
PH02Gene06165	RecName: Full=1,4-alpha-glucan-branching enzyme 1, chloroplastic/amyloplastic; AltName: Full=Starch branching enzyme I; Flags: Precursor	1.7136	-0.3379	-0.6493	-0.7265
PH02Gene13620	RecName: Full=Glucose-1-phosphate adenylyltransferase small subunit, chloroplastic/amyloplastic; AltName: Full=ADP-glucose pyrophosphorylase; AltName: Full=ADP-glu	1.7056	-0.6737	-0.2871	-0.7447
PH02Gene22653	RecName: Full=Beta-glucosidase 1; Short=Os1bglu1; Flags: Precursor	1.7032	-0.3619	-0.4857	-0.8556
PH02Gene25500	RecName: Full=Glucose-1-phosphate adenylyltransferase large subunit 1, chloroplastic/amyloplastic; AltName: Full=ADP-glucose pyrophosphorylase; AltName: Full=ADP-gl	1.7281	-0.4916	-0.5569	-0.6796
PH02Gene29656	RecName: Full=1,4-alpha-glucan-branching enzyme 2, chloroplastic/amyloplastic; AltName: Full=Q-enzyme; AltName: Full=Starch-branching enzyme IIB; Flags: Precursor	1.6540	-0.2667	-0.3540	-1.0333
PH02Gene37359	RecName: Full=Granule-bound starch synthase 1b, chloroplastic/amyloplastic; AltName: Full=Granule-bound starch synthase Ib; Flags: Precursor	1.6587	-0.2487	-0.3945	-1.0155
PH02Gene38601	RecName: Full=Beta-glucosidase A; AltName: Full=Beta-D-glucoside glucohydrolase A; AltName: Full=Cellobiase A; AltName: Full=Gentiobiase A; Flags: Precursor	1.6497	-0.1115	-0.5653	-0.9728
PH02Gene38723	RecName: Full=Alpha-1,4 glucan phosphorylase L-2 isozyme, chloroplastic/amyloplastic; AltName: Full=Starch phosphorylase L-2; Flags: Precursor	1.7085	-0.3942	-0.4814	-0.8329
PH02Gene44838	RecName: Full=Glucose-1-phosphate adenylyltransferase large subunit 1,	1.6503	-0.0865	-0.6299	-0.9339

	chloroplastic/amyloplastic; AltName: Full=ADP-glucose pyrophosphorylase; AltName: Full=ADP-gl				
PH02Gene02876	RecName: Full=Beta-amylase 3, chloroplastic; AltName: Full=1,4-alpha-D-glucan maltohydrolase; AltName: Full=Beta-amylase 8; AltName: Full=Chloroplast beta-amylase;	-1.2953	-0.3313	1.4802	0.1463
PH02Gene09572	RecName: Full=Sucrose synthase 5; Short=OsSUS5; AltName: Full=Sucrose-UDP glucosyltransferase 5	-1.3882	-0.2517	1.3966	0.2432
PH02Gene19719	RecName: Full=Sucrose synthase 7; Short=OsSUS7; AltName: Full=Sucrose-UDP glucosyltransferase 7	-1.5769	0.0608	1.1825	0.3337
PH02Gene04423	RecName: Full=Sucrose synthase 1; Short=OsSUS1; AltName: Full=Sucrose synthase 2; Short=RSs2; AltName: Full=Sucrose-UDP glucosyltransferase 1	-1.7233	0.7215	0.4373	0.5645
PH02Gene05641	RecName: Full=UTP--glucose-1-phosphate uridylyltransferase; AltName: Full=UDP-glucose pyrophosphorylase; Short=UDPGP; Short=UGPase	-1.7251	0.5176	0.4875	0.7201
PH02Gene09580	RecName: Full=Sucrose:sucrose 1-fructosyltransferase; AltName: Full=Sucrose 1(F)-fructosyltransferase; AltName: Full=Sucrose:sucrose 1(F)-beta-D-fructosyltransferase	-1.6580	0.0827	0.7454	0.8299
PH02Gene12539	RecName: Full=Endoglucanase 10; AltName: Full=Endo-1,4-beta glucanase 10; AltName: Full=OsGLU2	-1.7258	0.7114	0.4838	0.5305
PH02Gene14100	RecName: Full=Lysosomal beta glucosidase; Flags: Precursor	-1.7102	0.5218	0.3744	0.8141
PH02Gene14729	RecName: Full=Probable alpha,alpha-trehalose-phosphate synthase	-1.7115	0.3278	0.7467	0.6370
PH02Gene15089	RecName: Full=Probable alpha,alpha-trehalose-phosphate synthase	-1.6283	-0.0139	0.8257	0.8165
PH02Gene20325	RecName: Full=Probable trehalose-phosphate phosphatase 1; Short=OsTPP1; AltName: Full=Trehalose 6-phosphate phosphatase	-1.6977	0.2555	0.6425	0.7998
PH02Gene26446	RecName: Full=Beta-fructofuranosidase, insoluble isoenzyme 1; AltName: Full=Cell wall beta-fructosidase 1; AltName: Full=Invertase 1; AltName: Full=OsCIN1; AltName	-1.7248	0.6106	0.4313	0.6828
PH02Gene27917	RecName: Full=Sucrose synthase 7; Short=OsSUS7; AltName: Full=Sucrose-UDP glucosyltransferase 7	-1.6827	0.3571	0.9478	0.3778
PH02Gene29429	RecName: Full=Sucrose synthase 7; Short=OsSUS7; AltName: Full=Sucrose-UDP	-1.6544	0.0680	0.8025	0.7839

	glucosyltransferase 7				
PH02Gene43583	RecName: Full=Fructokinase-2; AltName: Full=Fructokinase II; AltName: Full=OsFKII	-1.6752	0.1876	0.5823	0.9053
PH02Gene44962	RecName: Full=Beta-glucosidase 25; Short=Os6bglu25; Flags: Precursor	-1.7267	0.4615	0.6826	0.5826
PH02Gene46034	RecName: Full=Sucrose synthase 1; Short=OsSUS1; AltName: Full=Sucrose synthase 2; Short=RSs2; AltName: Full=Sucrose-UDP glucosyltransferase 1	-1.6904	0.2550	0.5640	0.8715
PH02Gene04686	RecName: Full=Beta-glucosidase 31; Short=Os9bglu31; Flags: Precursor	-1.5126	-0.1998	0.5351	1.1772
PH02Gene05690	RecName: Full=Beta-fructofuranosidase 1; AltName: Full=Invertase 1; AltName: Full=Sucrose 1; Flags: Precursor	-1.5279	-0.2227	0.6713	1.0794
PH02Gene22525	RecName: Full=Probable alpha,alpha-trehalose-phosphate synthase	-1.1977	-0.7713	0.8584	1.1107
PH02Gene26917	RecName: Full=Probable trehalose-phosphate phosphatase 7; Short=OsTPP7; AltName: Full=Trehalose 6-phosphate phosphatase	-1.5229	-0.2629	0.7999	0.9859
PH02Gene30801	RecName: Full=Alpha,alpha-trehalose-phosphate synthase	-1.5951	-0.0212	0.5355	1.0809
PH02Gene33818	RecName: Full=Beta-amylase 1, chloroplastic; AltName: Full=1,4-alpha-D-glucan maltohydrolase; AltName: Full=Beta-amylase 7; AltName: Full=Thioredoxin-regulated beta	-1.4523	-0.2924	0.4960	1.2487
PH02Gene41803	RecName: Full=Beta-glucosidase 16; Short=Os4bglu16; Flags: Precursor	-1.4784	-0.3501	0.8137	1.0147
PH02Gene42897	RecName: Full=Probable alpha,alpha-trehalose-phosphate synthase	-1.4696	-0.3450	0.7143	1.1004
PH02Gene46771	RecName: Full=Probable trehalose-phosphate phosphatase 7; Short=OsTPP7; AltName: Full=Trehalose 6-phosphate phosphatase	-1.5282	-0.2592	0.8959	0.8915
PH02Gene06193	RecName: Full=Beta-fructofuranosidase, insoluble isoenzyme 2; AltName: Full=Cell wall beta-fructosidase 2; AltName: Full=Invertase 2; AltName: Full=OsCIN2; AltName	-1.6795	0.9195	0.2297	0.5302
PH02Gene19810	RecName: Full=Beta-glucosidase 6; Short=Os3bglu6; Flags: Precursor	-1.4761	1.3079	-0.1354	0.3036
PH02Gene27250	RecName: Full=Endoglucanase 10; AltName: Full=Endo-1,4-beta glucanase 10; AltName: Full=OsGLU2	-1.6451	0.9982	0.5335	0.1134
PH02Gene46133	RecName: Full=Hexokinase-6; AltName: Full=Hexokinase-2	-1.6491	0.8775	0.0597	0.7120
PH02Gene47637	RecName: Full=Beta-glucosidase 27; Short=Os8bglu27; Flags: Precursor	-1.6146	1.0084	-0.0071	0.6133
PH02Gene47638	RecName: Full=Beta-glucosidase 28; Short=Os8bglu28; Flags: Precursor	-1.5934	1.1711	0.2270	0.1952

PH02Gene06290	RecName: Full=Hexokinase-8; AltName: Full=Hexokinase-4	1.5426	-0.0753	-0.2150	-1.2523
PH02Gene09014	RecName: Full=Beta-glucosidase 25; Short=Os6bglu25; Flags: Precursor	1.5938	-0.4220	-0.0405	-1.1314
PH02Gene12545	RecName: Full=Glucose-1-phosphate adenylyltransferase large subunit 1, chloroplastic; AltName: Full=ADP-glucose pyrophosphorylase; AltName: Full=ADP-glucose syntha	1.5707	0.0796	-0.5382	-1.1122
PH02Gene14970	RecName: Full=1,4-alpha-glucan-branching enzyme 3, chloroplastic/amyloplastic; Short=AtSBE III; AltName: Full=Branching enzyme 1; Short=AtBE1; AltName: Full=Prote	1.5195	-0.0825	-0.1475	-1.2895
PH02Gene17166	RecName: Full=Glucose-1-phosphate adenylyltransferase large subunit 2, chloroplastic; AltName: Full=ADP-glucose pyrophosphorylase; AltName: Full=ADP-glucose syntha	1.4768	0.1410	-0.3136	-1.3042
PH02Gene17228	RecName: Full=Starch synthase 1, chloroplastic/amyloplastic; AltName: Full=Starch synthase I-2; Short=SS I; Short=SS I-2; Flags: Precursor	1.4939	0.0442	-0.2290	-1.3091
PH02Gene26491	RecName: Full=Sucrose synthase 4; Short=OsSUS4; AltName: Full=Sucrose-UDP glucosyltransferase 4	1.4996	-0.2092	0.0162	-1.3066
PH02Gene29455	RecName: Full=1,4-alpha-glucan-branching enzyme, chloroplastic/amyloplastic; AltName: Full=Q-enzyme; AltName: Full=Starch-branching enzyme; Flags: Precursor	1.2281	0.2208	0.1102	-1.5591
PH02Gene31595	RecName: Full=1,4-alpha-glucan-branching enzyme, chloroplastic/amyloplastic; AltName: Full=Q-enzyme; AltName: Full=Starch-branching enzyme; Flags: Precursor	1.4987	0.0141	-0.2043	-1.3084
PH02Gene44935	RecName: Full=Phosphoglucomutase, chloroplastic; Short=PGM; AltName: Full=Glucose phosphomutase; Flags: Precursor	1.5559	0.0433	-0.4133	-1.1859
PH02Gene11050	RecName: Full=Beta-amylase 1, chloroplastic; AltName: Full=1,4-alpha-D-glucan maltohydrolase; AltName: Full=Beta-amylase 7; AltName: Full=Thioredoxin-regulated beta	-1.6649	0.4144	0.2546	0.9958
PH02Gene13448	RecName: Full=Glucose-6-phosphate isomerase, cytosolic; Short=GPI; AltName: Full=Phosphoglucose isomerase; Short=PGI; AltName: Full=Phosphohexose isomerase; Short=PH	-1.6363	0.4109	0.1638	1.0616
PH02Gene20078	RecName: Full=Sucrose:sucrose 1-fructosyltransferase; AltName: Full=Sucrose 1(F)-fructosyltransferase; AltName: Full=Sucrose:sucrose 1(F)-beta-D-fructosyltransferase	-1.6568	0.3025	0.3262	1.0281
PH02Gene24831	RecName: Full=Hexokinase-5; AltName: Full=Hexokinase I	-1.5935	0.6864	-0.0840	0.9912

PH02Gene27125	RecName: Full=Fructokinase-1; AltName: Full=Fructokinase I; AltName: Full=OsFKI	-1.6656	0.3489	0.3141	1.0027
PH02Gene31459	RecName: Full=Probable trehalose-phosphate phosphatase 6; Short=OsTPP6; AltName: Full=Trehalose 6-phosphate phosphatase	-1.6619	0.3374	0.3107	1.0138
PH02Gene37603	RecName: Full=Fructokinase-2; AltName: Full=Fructokinase II; AltName: Full=OsFKII	-1.6749	0.4127	0.2936	0.9686
PH02Gene45438	RecName: Full=Hexokinase-7; AltName: Full=Hexokinase-6	-1.5557	0.2258	0.0972	1.2326
PH02Gene26251	RecName: Full=Probable trehalase; AltName: Full=Alpha,alpha-trehalase; AltName: Full=Alpha,alpha-trehalose glucohydrolase	-1.0635	0.9789	1.0188	-0.9342
PH02Gene37127	RecName: Full=Alpha-amylase; AltName: Full=1,4-alpha-D-glucan glucanohydrolase; AltName: Full=Alpha-amylase isozyme 1B; Flags: Precursor	-1.3011	1.0523	0.8889	-0.6401
PH02Gene30298	RecName: Full=UTP--glucose-1-phosphate uridylyltransferase; AltName: Full=UDP-glucose pyrophosphorylase; Short=UDPGP; Short=UGPase	-1.4300	-0.1435	0.1967	1.3769
PH02Gene32320	RecName: Full=Beta-amylase 3, chloroplastic; AltName: Full=1,4-alpha-D-glucan maltohydrolase; AltName: Full=Beta-amylase 8; AltName: Full=Chloroplast beta-amylase;	-1.3938	-0.1313	0.1005	1.4247
PH02Gene47723	RecName: Full=Sucrose synthase 7; Short=OsSUS7; AltName: Full=Sucrose-UDP glucosyltransferase 7	-1.2869	-0.4207	0.2586	1.4491
PH02Gene40204	_	-1.3790	0.3149	1.3785	-0.3144
PH02Gene47722	RecName: Full=Sucrose synthase 7; Short=OsSUS7; AltName: Full=Sucrose-UDP glucosyltransferase 7	-1.6349	0.6313	0.9628	0.0409

Note:(1)description: name of gene code. (2)There are four growth and development stages of bamboo shoots: the expression level after standardization.

Table S6 Co-expression network of DEGs at the top of Moso bamboo shoots

gene id	degree	description
PH02Gene26491	15.54354	RecName: Full=Sucrose synthase 4; Short=OsSUS4; AltName: Full=Sucrose-UDP glucosyltransferase 4
PH02Gene07829	15.03386	RecName: Full=Probable sucrose-phosphate synthase 4; AltName: Full=Sucrose phosphate synthase 4F; Short=OsSPS4F; AltName: Full=UDP-glucose-fructose-phosphate gluco
PH02Gene01052	14.97322	RecName: Full=Soluble starch synthase 2-2, chloroplastic/amyloplastic; AltName: Full=Soluble starch synthase II-2; Flags: Precursor
PH02Gene48441	14.91505	RecName: Full=Sucrose synthase 4; Short=OsSUS4; AltName: Full=Sucrose-UDP glucosyltransferase 4
PH02Gene13620	14.41779	RecName: Full=Glucose-1-phosphate adenylyltransferase small subunit, chloroplastic/amyloplastic; AltName: Full=ADP-glucose pyrophosphorylase; AltName: Full=ADP-glu
PH02Gene17166	14.16257	RecName: Full=Glucose-1-phosphate adenylyltransferase large subunit 2, chloroplastic; AltName: Full=ADP-glucose pyrophosphorylase; AltName: Full=ADP-glucose syntha
PH02Gene12545	13.99325	RecName: Full=Glucose-1-phosphate adenylyltransferase large subunit 1, chloroplastic; AltName: Full=ADP-glucose pyrophosphorylase; AltName: Full=ADP-glucose syntha
PH02Gene39746	13.91867	RecName: Full=Probable inactive beta-glucosidase 14; Short=Os4bglu14; Flags: Precursor
PH02Gene01266	13.79487	RecName: Full=Isoamylase 3, chloroplastic; Short=AtISA3; Flags: Precursor
PH02Gene38601	13.55319	RecName: Full=Beta-glucosidase A; AltName: Full=Beta-D-glucoside glucohydrolase A; AltName: Full=Cellobiase A; AltName: Full=Gentiobiase A; Flags: Precursor
PH02Gene13763	13.42544	RecName: Full=Alpha,alpha-trehalose-phosphate synthase

PH02Gene06165	13.40999	RecName: Full=1,4-alpha-glucan-branching enzyme 1, chloroplastic/amyloplastic; AltName: Full=Starch branching enzyme I; Flags: Precursor
PH02Gene44838	13.19952	RecName: Full=Glucose-1-phosphate adenylyltransferase large subunit 1, chloroplastic/amyloplastic; AltName: Full=ADP-glucose pyrophosphorylase; AltName: Full=ADP-gl
PH02Gene18798	13.12174	RecName: Full=Probable alpha,alpha-trehalose-phosphate synthase
PH02Gene14465	12.72853	RecName: Full=Nudix hydrolase 14, chloroplastic; Short=AtNUDT14; AltName: Full=ADP-sugar diphosphatase; Short=AtASPP; Flags: Precursor

Note:(1) degree：Node connectivity. The larger the degree, the more important it is(2) description:Name of gene code

Table S7 Co-expression network of DEGs at the base of Moso bamboo shoots

gene id	degree	description
PH02Gene01266	14.90276	RecName: Full=Isoamylase 3, chloroplastic; Short=AtISA3; Flags: Precursor
PH02Gene37359	14.69078	RecName: Full=Granule-bound starch synthase 1b, chloroplastic/amyloplastic; AltName: Full=Granule-bound starch synthase Ib; Flags: Precursor
PH02Gene29656	14.03499	RecName: Full=1,4-alpha-glucan-branching enzyme 2, chloroplastic/amyloplastic; AltName: Full=Q-enzyme; AltName: Full=Starch-branching enzyme IIB; Flags: Precursor
PH02Gene01052	13.97282	RecName: Full=Soluble starch synthase 2-2, chloroplastic/amyloplastic; AltName: Full=Soluble starch synthase II-2; Flags: Precursor
PH02Gene31595	13.81575	RecName: Full=1,4-alpha-glucan-branching enzyme, chloroplastic/amyloplastic; AltName: Full=Q-enzyme; AltName: Full=Starch-branching enzyme; Flags: Precursor
PH02Gene06290	13.75796	RecName: Full=Hexokinase-8; AltName: Full=Hexokinase-4
PH02Gene26491	13.37804	RecName: Full=Sucrose synthase 4; Short=OsSUS4; AltName: Full=Sucrose-UDP glucosyltransferase 4
PH02Gene14970	13.20047	RecName: Full=1,4-alpha-glucan-branching enzyme 3, chloroplastic/amyloplastic; Short=AtSBE III; AltName: Full=Branching enzyme 1; Short=AtBE1; AltName: Full=Prote

PH02Gene06165	13.00712	RecName: Full=1,4-alpha-glucan-branching enzyme 1, chloroplastic/amyloplastic; AltName: Full=Starch branching enzyme I; Flags: Precursor
PH02Gene17228	12.77285	RecName: Full=Starch synthase 1, chloroplastic/amyloplastic; AltName: Full=Starch synthase I-2; Short=SS I; Short=SS I-2; Flags: Precursor
PH02Gene38723	12.54154	RecName: Full=Alpha-1,4 glucan phosphorylase L-2 isozyme, chloroplastic/amyloplastic; AltName: Full=Starch phosphorylase L-2; Flags: Precursor
PH02Gene12545	12.43129	RecName: Full=Glucose-1-phosphate adenylyltransferase large subunit 1, chloroplastic; AltName: Full=ADP-glucose pyrophosphorylase; AltName: Full=ADP-glucose syntha
PH02Gene44838	12.40378	RecName: Full=Glucose-1-phosphate adenylyltransferase large subunit 1, chloroplastic/amyloplastic; AltName: Full=ADP-glucose pyrophosphorylase; AltName: Full=ADP-gl
PH02Gene05928	12.18266	RecName: Full=Isoamylase 3, chloroplastic; Short=AtISA3; Flags: Precursor
PH02Gene38601	12.10717	RecName: Full=Beta-glucosidase A; AltName: Full=Beta-D-glucoside glucohydrolase A; AltName: Full=Cellobiase A; AltName: Full=Gentiobiase A; Flags: Precursor

Note:(1) degree：Node connectivity. The larger the degree, the more important it is(2) description:Name of gene code

Table S8 Sequences of qRT-PCR primers used in this study

Target gene	Primer	5'----3'
PH02Gene04423	PH02Gene04423-F	TGCGGTCTTCCGACATTC
	PH02Gene04423-R	GGGTCTTGCTGGCACTTA
PH02Gene46034	PH02Gene46034-F	AGGGTGGGCTTCAGCGTAT
	PH02Gene46034-R	AGGTAGCGGCGGGTTTCA
PH02Gene06165	PH02Gene06165-F	TGAAGCGAGCACAACAGT
	PH02Gene06165-R	TCTTTCCAGCATAGGGTC
PH02Gene11050	PH02Gene11050-F	CGAAATGGGTATCGGAGGAGA
	PH02Gene11050-R	CGCATGAAGTCGGCGTAGCA
Internal control gene	ACT-F	TACGCTTCCTCACGCTATTCTTC
	ACT-R	TTCTCGCTCGGCAGTTGTTG
