# Supplementary material for: Association among starch storage, metabolism, related genes and growth of Moso bamboo (Phyllostachys heterocycla) shoots
Source: BMC Plant Biol. 2021 Oct 20;21:477. doi: 10.1186/s12870-021-03257-2 (PMC8527747; doi:10.1186/s12870-021-03257-2)
Supplement: Supplementary file 2 — Additional file 2: Figure S1 GO functional annotation analysis of DEGs at the top (left) and base (right) of Moso bamboo shoots. [file 12870_2021_3257_MOESM2_ESM.pdf]

Supplementary File 2

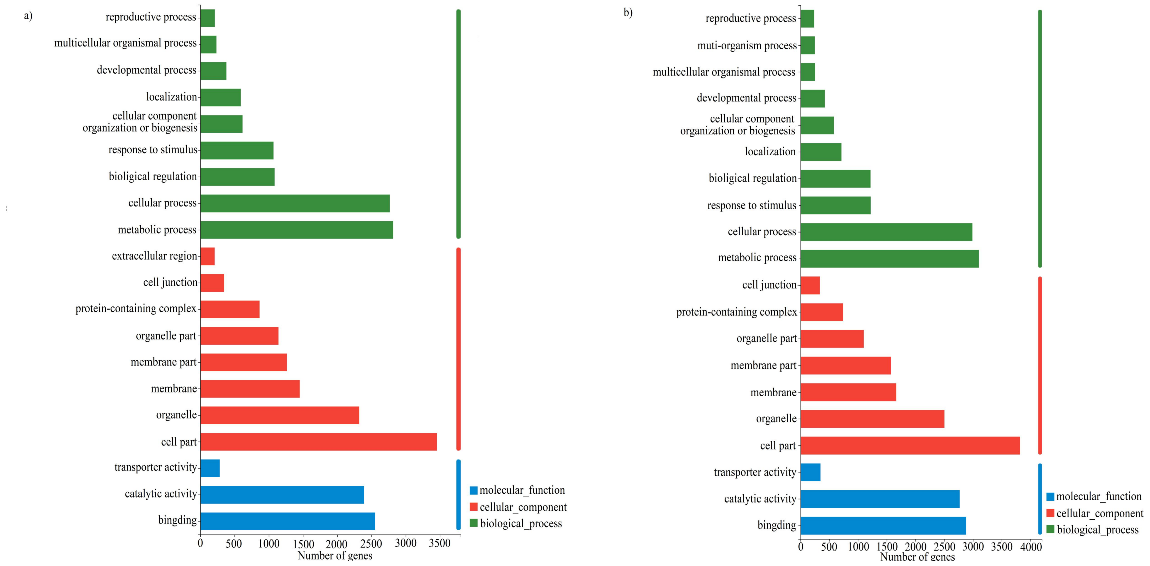

Figure S1 GO functional annotation analysis at DEGs in the top (left) and base (right) of Moso bamboo shoots. The vertical axis represents the terms of the secondary classification of GO, the horizontal axis represents the number of genes compared to the secondary classification, and the three colors represent the three classifications.
